# Changes in primary visual and auditory cortex of blind and sighted adults following 10 weeks of click-based echolocation training

**DOI:** 10.1093/cercor/bhae239

**Published:** 2024-06-20

**Authors:** Liam J Norman, Tom Hartley, Lore Thaler

**Affiliations:** Department of Psychology, Durham University, Durham, DH1 3LE, UK; Department of Psychology and York Biomedical Research Institute, University of York, Heslington, YO10 5DD, UK; Department of Psychology, Durham University, Durham, DH1 3LE, UK

**Keywords:** blindness, audition, neuroplasticity, fMRI

## Abstract

Recent work suggests that the adult human brain is very adaptable when it comes to sensory processing. In this context, it has also been suggested that structural “blueprints” may fundamentally constrain neuroplastic change, e.g. in response to sensory deprivation. Here, we trained 12 blind participants and 14 sighted participants in echolocation over a 10-week period, and used MRI in a pre–post design to measure functional and structural brain changes. We found that blind participants and sighted participants together showed a training-induced increase in activation in left and right V1 in response to echoes, a finding difficult to reconcile with the view that sensory cortex is strictly organized by modality. Further, blind participants and sighted participants showed a training induced increase in activation in right A1 in response to sounds per se (i.e. not echo-specific), and this was accompanied by an increase in gray matter density in right A1 in blind participants and in adjacent acoustic areas in sighted participants. The similarity in functional results between sighted participants and blind participants is consistent with the idea that reorganization may be governed by similar principles in the two groups, yet our structural analyses also showed differences between the groups suggesting that a more nuanced view may be required.

## Introduction

In the last 25 years, neuroscience has begun to acknowledge the capacity of sensory areas to exhibit a striking degree of plasticity (see Cecchetti et al. 2016 and Amedi et al. 2017 for reviews). Recently, it has been proposed that brain plasticity in the context of changes in sensory input or training may be fundamentally constrained by an inherent “blueprint” of structural connections in the brain (Makin and Krakauer 2023). A prediction of this framework would be that similar neuroplastic change should be observed in response to training in people with and without long-term sensory deprivation. Brain plasticity of similar form in adults with and without sensory loss has now been observed in higher-order sensory areas in response to training, e.g. in which people learn novel sensory skills over a period of several days, weeks, or months (e.g. Amedi et al. 2007; Ptito et al. 2009; Reich et al. 2011; Striem-Amit et al. 2012; Siuda-Krzywicka et al. 2016; Aggius-Vella et al. 2023). Similar findings for primary sensory areas are lacking, however, in particular for studies with crucial pre–post training measurements. A recent study (Aggius-Vella et al. 2023), for example, used a pre–post design and trained both blind and sighted people to navigate using a visual-to-auditory substitution device over 4 days. They found that people who were blind from birth showed increased activity in area V6 (measured with functional magnetic resonance imaging [fMRI]) in response to auditory navigation after 3 days of training. This activity had not been present before training. The authors found no change in response in A1 and did not report on any changes in V1. In an earlier related study, Maidenbaum et al. (2018) observed an increased response in V1 in sighted people following the exact same training, but the implications of that finding are ambiguous due to the fMRI response in V1 not rising above zero after training.

In sum, although there is some evidence that V1 can be recruited for processing of sensory input in different modalities in the early blind (e.g. Sadato et al. 1996; Ptito et al. 2008b; Kupers et al. 2010; Bedny et al. 2011; Thaler et al. 2011; Norman and Thaler 2019), it is an open question if this kind of functional plasticity in primary sensory areas can be considered a “normal” property of the adult human brain or whether it is dependent on long-term sensory deprivation.

Thus, to address this question, here we used short-term (i.e. 10-week) training in click-based echolocation in combination with magnetic resonance imaging (MRI) to investigate effects of training on function and structure in A1 and V1 in people with typical vision and in people with long-term visual deprivation (i.e. blindness).

Echolocation is the ability to perceive the spatial environment through sound echoes (Griffin 1944), and it is now well documented that blind and sighted people can learn this skill (e.g. Teng and Whitney 2011; Tonelli et al. 2016; Dodsworth et al. 2020; Norman et al. 2021; for reviews, see Kolarik et al. 2014, 2021; Thaler and Goodale 2016). In people who are blind and who have long-term experience in echolocation (i.e. 10 years or more of daily use; while the majority will have had experience early in development, there are also some who had experience only later), it has been shown that echolocation recruits not only early auditory areas including A1 but also early visual cortex including V1 (e.g. Thaler et al. 2011; Wallmeier et al. 2015; Flanagin et al. 2017; Norman and Thaler 2019). The effects of short-term training have not been studied, and there have been no direct comparisons between trained blind and sighted people. Thus, here, we investigate whether these plasticity principles extend to blind and sighted people who complete short-term training in echolocation in adulthood.

We took functional and structural MRI measurements in 12 blind and 14 sighted participants before and after they were trained in click-based echolocation over a 10-week period (20 sessions, each 2–3 h in length). Participants were trained in three different tasks (size discrimination, orientation perception, virtual navigation) and also navigated using echolocation in natural environments. The training program and behavioral performance of these participants on these tasks over the training program has been described in detail previously (Norman et al. 2021). The fMRI task we used was an echo-acoustic spatial navigation task introduced by Norman and Thaler (2023). This task allowed us to separately investigate effects of learning on brain activation for three different aspects of processing. Specifically, processing of (i) sound per se (i.e. listening to sound vs. silence), (ii) echoes per se (i.e. listening to sound with echoes vs. sound without echoes), and (iii) spatiotemporal echo-acoustic information (i.e. listening to sound with spatiotemporally coherent echo information vs. sound without spatiotemporally coherent echo information). We used a region-of-interest (ROI) approach, focusing on V1 and A1, and treating left and right hemispheres separately, as previous studies suggest a potential right-lateralized preference for echolocation processing (Thaler et al. 2011; Fiehler et al. 2015). In addition, we included the occipital place area (OPA), because expert echolocators who are blind show increased activity in OPA for processing of echo-acoustic spatially coherent information (Norman and Thaler 2023), and as such, we may observe functional recruitment after training. Using the same ROIs, we also ran a longitudinal analysis of changes in gray matter density (using voxel-based morphometry; VBM) in the blind and sighted participants.

## Materials and methods

### Ethics

All procedures followed the British Psychological Society code of practice and the World Medical Association’s Declaration of Helsinki. The experiment had received ethical approval by the Ethics Advisory Sub-Committee in the Department of Psychology at Durham University (Ref 14/13). All participants gave written informed consent to take part in this study. All forms were provided in preferred accessible format to all blind participants (i.e. braille, audio file, or electronic format for screen reader). Participants who were sighted and participants who were blind received £6/hr and £10/hr, respectively, to compensate them for their effort and time taking part.

### Participants

Participants were recruited via opportunity sampling. We tested both blind and adult sighted participants (BPs and SPs, respectively), all with no prior experience in click-based echolocation. Details of our BP sample (6 males, 6 females) are shown in Table 1. At the time of testing, all BPs had a profound level of blindness, with 8 out of the 12 participants being either totally blind or having only bright light detection, and the remaining 4 participants having no form or spatial vision. For 11 out of 12 BPs, the *onset* of vision loss was at birth, and for 1, the onset had been at 3 months of age. For BPs where the age at onset differed from the age at official diagnosis/certification as blind, we have indicated this in Table 1. Please note that for two BPs (BP3 and BP9), while vision loss had been present from birth, their official diagnosis/certification as blind occurred at a later age that might have coincided with onset of puberty or shortly after (i.e. 10 and 13 yr), and indicating that they were diagnosed/certified as “vision impaired” (but not “blind”) before that age. In sum, all our BPs were profoundly blind, and the majority were also early blind. All our BPs were independent travelers, and all had received mobility and orientation training as part of visual impairment (VI) habilitation and VI rehabilitation that is provided to people with VI in the United Kingdom.

**Table 1:** Details of BPs. ^*^Please note that for BPs where the age at onset of vision loss differed from the age at official diagnosis/certification as blind, we provide information for both.

Participant	Sex	Age	Degree of vision loss	Cause and age at onset of vision loss^*^	Echolocation use
BP1	F	60	Total blindness left eye; bright light detection right eye.	Stichler’s syndrome. Retinal sciasis, from birth.	Some experience; very little regular use
BP2	M	38	Decreased field of view (<2 deg) and decreased acuity (<20/200) in both eyes. No form or spatial vision.	Retinitis pigmentosa and other retinal pathology (unknown), from birth. Official diagnosis/certification as blind in early childhood (no exact age remembered but was known when commencing school, i.e. age 5 yr).	None
BP3	M	54	Bright light detection	Retinitis pigmentosa, from birth. Official diagnosis/certification as blind at age 10 yr	Some experience; very little regular use
BP4	M	39	Bright light detection	Retinitis pigmentosa, from birth. Official diagnosis/certification as blind in early childhood (no exact age remembered but was known when commencing school, i.e. age 5 yr).	None
BP5	F	44	Total blindness right eye; bright light detection left eye.	Micropthalmia and glaucoma, from birth; right eye enucleated aged 39 yr	None
BP6	F	72	Bright light detection.	Retinitis pigmentosa. from birth. Official diagnosis/certification as blind in early childhood (no exact age remembered but was known when commencing school, i.e. age 5 yr).	None
BP7	M	46	Total blindness	Ocular albinism, from birth.	Some experience; very little regular use
BP8	F	36	Bright light detection.	Unknown cause, from birth.	None
BP9	M	37	Decreased field of view (<5 deg) and decreased acuity (<20/200) in both eyes. No form or spatial vision.	Retinitis pigmentosa, from birth. Official diagnosis/certification as blind at age 13 yr.	None
BP10	F	27	Decreased field of view (1 deg) and decreased acuity (<20/200) left eye; bright light detection right eye. No form or spatial vision.	Leber’s amaurosis and cataracts, from birth.	None
BP11	F	79	Decreased field of view (“foveal”) and decreased acuity (<20/200) both eyes. No form or spatial vision.	Rod cone dystrophy, from birth.	None
BP12	M	48	Total blindness left eye; bright light detection right eye.	Severe childhood glaucoma, from 3 months old.	None

SPs (8 males, 6 females; ages: 21, 21, 22, 22, 23, 24, 25, 27, 32, 35, 38, 48, 60, and 71; mean = 33.5, SD = 15.8, median = 26) all reported to have normal or corrected to normal vision (based on self-report).

With the exception of one blind participant (BP6, aged 72 yr who wore hearing aids to compensate for age-related hearing loss), all participants had normal hearing appropriate for their age group (ISO 7029:2017) assessed using pure tone audiometry. For purposes of testing, the participant with hearing aids did not wear their aids during any of the experimental testing sessions, as they would not be able to wear these in the MRI scanner. All participants with any ability to sense light (BP as well as SP) wore a blindfold.

All BPs and SPs took part in two MRI sessions to measure brain activity and structure before and after a 10-week echolocation training program.

### 10-week echolocation training program

**Fig. 1:**
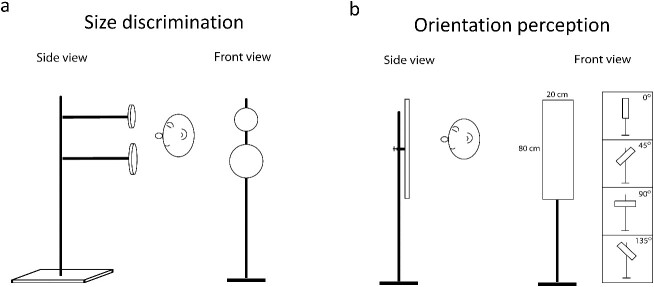
Practical echolocation tasks used in the 10-week training program. In the size discrimination task (a), participants judged which of two vertically arranged disks was larger. In the orientation perception task (b), participants judged whether the rectangular plank was vertical, right side up (45°), left side up (135°), or horizontal.

**Fig. 2:**
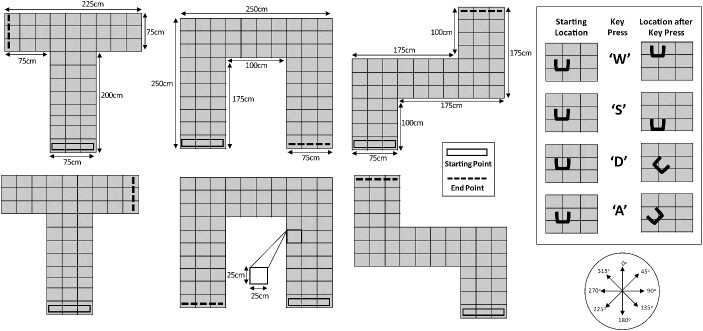
Virtual echo-acoustic navigation task used in the 10-week training program. Top-down illustrations of the spatial arrangements of each maze are shown. Participants used the computer keyboard (inset on the right-hand side) to move from the starting area (black box outline) to the end goal (dashed black line), which was constructed from a different material to the other walls.

BPs and SPs completed a 10-week echolocation training program consisting of 20 sessions of practical and computer-based echolocation tasks. These tasks have been described in detail in a previous paper (Norman et al. 2021). To summarize briefly here, each session included a size discrimination task (Fig. 1a), an orientation perception task (Fig. 1b), and a virtual echo-acoustic maze navigation task (Fig. 2) constructed from echolocation sounds recorded in real physical spaces. In addition, each session also included a component in which participants used click-based echolocation to explore real indoor and outdoor environments using echolocation, under the guidance of an experimenter. Participants spent 2 h on each training session, during which the time spent on the four tasks was distributed fairly equally. This varied somewhat across participants and sessions depending on how quickly they finished certain tasks. The behavioral task used in the current study was separate from the four training tasks, as it instead served as a fixed measure of performance once before the 10-week training and once after.

### fMRI (pre- and post-training)

BPs and SPs each completed the same fMRI-based echolocation task twice: once before and once after completing the training. The task required participants to listen to prerecorded binaural echolocation sounds (i.e. echo-acoustic sound through a first-person perspective) and to make perceptual judgments about them. Prior to each fMRI session, participants also completed this same task outside the scanner. The sounds and task used during and before MRI scanning have been described in detail elsewhere (Norman and Thaler 2023), but see a brief description below.

#### Echolocation stimuli

The stimuli were created from a large set of recordings first described by Dodsworth et al. (2020). For full details of those stimuli, please refer to that report. Briefly, binaural recordings of clicks and click-echoes were made with an anthropometric manikin in physical spaces comprising corridors in specific spatial arrangements (T-mazes, U-mazes, Z-mazes). In addition, we also created spatially mirrored versions of these recordings, giving six maze layouts in total.

**Fig. 3:**
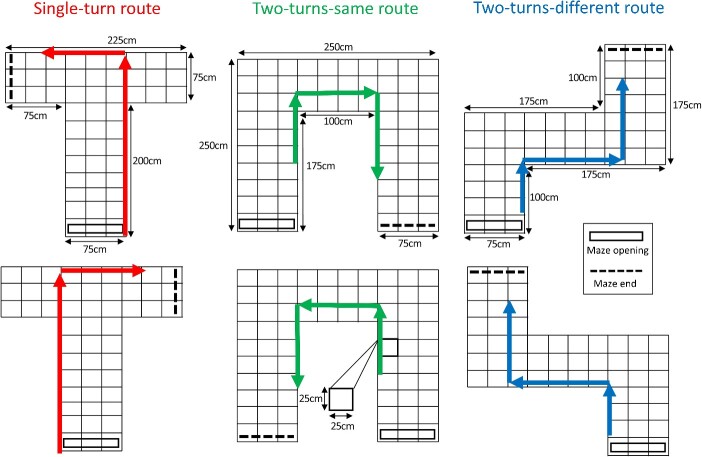
Illustration of spatial arrangements used to construct virtual spaces (T-mazes, U-mazes, Z-mazes) and the prespecified routes taken through each one. Each route was composed of 18 click recordings taken at regularly spaced intervals. Specifically, there was one click for each position along the route (marked by the intersections) and two clicks for each rotation of 90° (in 45° steps).

For each of the six mazes, we created two samples by selecting recordings corresponding to a specific sequence of locations and orientations within that maze (see Fig. 3). This gave a total of 12 sound files that were each 10.53 s in length and contained 18 clicks and echoes, each separated by 600 ms (a rate of 1.71 clicks/s). These 12 sound files were assigned to one of three categories: (i) single-turn route, (ii) two-turn route in same direction, and (iii) two-turn route in different (opposite) directions.

In addition to these spatially coherent route sounds, we created two types of control sounds: scrambled route sounds and clicks with no echoes. A scrambled route sound was created for each of the original route sounds in order to create sounds that had exactly the same low-level acoustic information (i.e. timing, clicks, and echoes), but did not convey spatially coherent information. To do this, the individual click-echo sounds in each route sound file were randomly shuffled and pieced together (maintaining the same click rate) so that there was no coherent route. In order to create a secondary set of control stimuli (i.e. stimuli with clicks but not containing any echoes), a sound recording was used during which the manikin had been placed facing the foam padded wall in the anechoic chamber. The sound was then repeated at the same temporal sequence as that for the “route” and “scrambled” sound files.

In total, five types of sound stimuli were created: single-turn route, two-turns-same route, two-turns-different route, scrambled route, and click only. Examples for each of these stimuli (in wav format) can be found on Open Science Framework: https://osf.io/c5pn2/, but note that playback of these example sounds should be done using a high-spec sound card and headphones, due to the nature of the echolocation sounds.

Stimuli containing echoes (i.e. “route” and “scrambled” stimuli) were of higher RMS intensity (specifically: T and T-scrambled: −41.4 dB; U and U scrambled: −41.4 dB; Z and Z-scrambled: −40.8 dB) than stimuli not containing echoes (i.e. “clicks”; −44.2 dB). In terms of absolute intensity at which sounds were played, each participant selected a sound intensity that felt comfortable for them to do the task. The same intensity was maintained for that participant throughout testing. Recorded sound files were filtered to achieve frequency response equalization for playback through the MRI-compatible insert earphones we used.

#### Experimental task outside the scanner

On a separate day before each fMRI session (pre- and post-training), participants completed two runs of 30 trials. On each trial, they heard one of the sound stimuli from one of the five categories (single-turn route, two-turns-same route, two-turns-different route, scrambled, and clicks only), with each condition being repeated six times. The order of trials was randomly determined at the start of each run. When the sound finished playing, participants gave a verbal response to indicate which category the sound belonged to. The experimenter recorded this response and started the next trial. Before participants performed the two runs of 30 trials, they were played two examples for each type of sound to make them familiar with the sounds and the required responses.

Participants completed the task in a sound-insulated and echo-acoustic dampened room (approx. 2.9 m × 4.2 m × 4.9 m) lined with foam wedges (cut-off frequency 315 Hz) in the Department of Psychology at Durham University. Sounds were played through MRI-compatible insert earphones (Model S-14, Sensimetrics, Malden, MA) encased in disposable foam tips (the earphones provided a 20- to 40-dB attenuation-level information). These earphones were amplified by a Kramer 900 N Stereo Power Amplifier (Kramer Electronics Ltd, Jerusalem, Israel), with input provided by a USB Soundcard (Creative Sound Blaster X-Fi HD Sound Card; Creative Technology Ltd, Creative Labs Ireland, Dublin, Ireland). The experimenter used a laptop (Dell Latitude E7470; Intel Core i56300U CPU 2.40; 8GB RAM; 64-bit Windows 7 Enterprise) running MATLAB R2018b (The Mathworks, Natick, MA, USA) and modified functions from the Psychtoolbox library (Brainard 1997) to control sound playback and to record participants’ responses.

#### Experimental task during fMRI scanning

Participants’ task inside the scanner was the same as that outside the scanner, with the following modifications. Participants gave their response after each stimulus presentation by pressing one of five buttons on an MR-compatible response unit. Each finger was assigned a different response (thumb = clicks only, index = single-turn, middle = two-turns-same, ring = two-turns-different, pinkie = scrambled). Participants were made familiar with the responses before commencing testing and were asked to press buttons corresponding to the various response categories via verbal prompt (i.e. without listening to stimuli). None of our participants reported any confusion or problems with the button responses. A beep (1.2 kHz, 50 ms) at the end of stimulus presentation prompted participants to respond. In addition to the five stimulus categories, a sixth “silence” category was also used (to allow comparisons to baseline activity in the fMRI data analysis). During these silence trials, no sound was played to participants and participants were told not to press a button. Thus, instructions were specific for all conditions, i.e. participants were told not to press a button after a silent trial, while they were told to press specific buttons after sound trials. Crucially, instructions were the same in pre- and post-training sessions. The order of stimulus presentation was counterbalanced with respect to the three main stimulus conditions (route, scrambled, and clicks only). This was achieved by breaking down 36 trials in each run into nine sequential groups of four. The first trial in each group was always a silence trial, and the remaining three were a random order of route, scrambled, and clicks only. The order of these three trial types was counterbalanced such that after every two runs, each type was presented equally often in each of the three sequence positions. The same randomized order of sounds was used for all participants, and this same order was used in the post- and pretraining scanning sessions.

For sound presentation, the same equipment as that used before fMRI scanning was used to play sounds, with the exception that a PC (Intel Core i7-6700 CPU 3.40; 8GB RAM; 64-bit Windows 7 Enterprise) was used instead of a laptop. Further, participants gave their response using an MRI-compatible 5-button fiber-optic button response unit (5-Button Fiber Optic Response Button System, Psychology Software Tools, Inc, Pittsburgh, USA) with their right hand. To minimize background noise, the MRI bore’s circulatory air fan was turned off during experimental runs. To minimize interference from light sources, all lights inside the MRI room were turned off and all participants with any ability to sense light (BP as well as SP) wore a blindfold. A sparse sampling imaging sequence was used (see below for details) to minimize scanner noise during auditory stimulus presentation.

#### fMRI scanning parameters

**Fig. 4:**
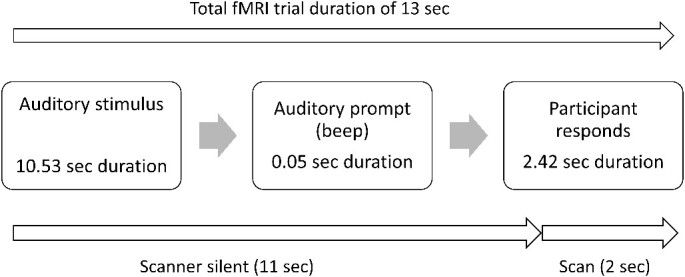
Illustration of fMRI trial sequence. Sound playback in each trial lasted 10.53 s, followed by a 50-ms beep that signaled the participant to make a response. Participants then had a 2.42-s window in which to respond, before the beginning of the next stimulus. The scanner was silent during the presentation of the sound (11 s), and volume acquisition took 2 s, overlapping the button response window.

All MRI data were acquired at Durham University Centre for Imaging (DCI, James Cook University Hospital, Middlesbrough, UK), with a 3-Tesla, whole-body MRI system (Magnetom Tim Trio; Siemens, Erlangen, Germany) and 32-channel head coil. High-resolution structural images for each participant were acquired using a T1-weighted magnetization-prepared, rapid-acquisition gradient echo (MPRAGE) sequence, at a resolution of 1 × 1 × 1 mm. Functional images were acquired using a single-shot gradient echo-planar pulse sequence in combination with a sparse sampling design (Hall et al. 1999), with a repetition time of 13 s (11-s inactivity for stimulus presentation, followed by 2 s of volume acquisition; see Fig. 4 for illustration). Thus, during stimulus presentation, no functional volumes were acquired. Instead, a single functional volume was acquired in the 2-s period after the end of stimulus presentation. The field of view was 192 mm with a matrix size of 64 × 64, giving an in-slice resolution of 3 mm. 38 contiguous axial slices were acquired in ascending order with a slice thickness of 3.5 mm, covering the whole brain. The echo time was 30 ms, and the flip angle was 90°. For each run, a total of 38 functional volumes were acquired, with each run lasting 8 min and 14 s. The first and last volume in each run were acquired after silence. A total of six runs were completed per participant in both the pre- and post-training sessions, except for one participant (BP2) where only four runs were completed in the pretraining session.

#### fMRI data processing

FMRI data preprocessing and analysis were carried out using FEAT (FMRI Expert Analysis Tool) Version 6.00, part of FSL (FMRIB’s Software Library, www.fmrib.ox.ac.uk/fsl; Woolrich et al. 2001; Woolrich et al. 2004).

Images were brain-extracted (using BET; Smith 2002), and within-participant registration of low-resolution functional images to high-resolution structural (T1) images was achieved using FLIRT (6 d.f. Jenkinson and Smith 2001; Jenkinson et al. 2002). Further nonlinear registration to MNI152 standard space (voxel size of 2 mm) was achieved using FNIRT (Andersson et al. 2007) with a warp resolution of 2 mm. The very first functional volume within each run was discarded, leaving 37 volumes to analyze, the first and last of which were acquired after silence. The following prestatistic processing was applied to each run of functional data: slice-timing correction using Hanning-windowed sinc interpolation, motion correction using MCFLIRT (Jenkinson et al. 2002), high-pass temporal filtering (maximum allowed period = 100 s, or 0.01 Hz), and—for the whole brain analysis—spatial smoothing (full-width at half maximum Gaussian kernel of 12 mm).

#### fMRI modeling and contrasts

In the first-level analysis for each run, three explanatory variables (EVs) were modeled using stick function regressors (with no hemodynamic response convolution, due to the sparse sampling design): route stimulus, scrambled stimulus, and no-echo stimulus. The silence trials were used as an implicit baseline. These EVs were then used to define the three contrasts of interest: route vs. scrambled (EV weights: route = +1, scrambled = −1, no echo = 0), echo vs. no echo (EV weights: route = +1, scrambled = +1, no echo = −2), and sound vs. silence (EV weights: route = +1, scrambled = +1, no echo = +1).

In a second-level analysis stage, single-participant activations for each contrast (averaged across runs) were calculated separately for the pre- and post-training sessions using a fixed effects model, by forcing the random effects variance to zero in FLAME (FMRIB’s Local Analysis of Mixed Effects; Beckmann et al. 2003, Woolrich et al. 2004, Woolrich 2008). The resulting contrast images (52 in total = 26 participants × 2 timepoints) were used in the ROI analysis (described below). In addition, to determine the nature of any effects underlying the contrast results, we analyzed the response to each of the three individual stimulus conditions (i.e. relative to silence baseline) in the same ROIs.

#### ROI definition and analysis

Three ROIs were defined in standard MNI space (see Table 2). Contrasts analyzed for each ROI were (i) sound vs. silence, (ii) echo vs. no-echo, and (iii) route vs. scrambled. FSL’s Featquery was used to extract percent signal change (PSC) associated with each of the three contrasts for each ROI for each participant.

**Table 2:** Details of the ROIs. For each named ROI, data were extracted separately for the left and right hemispheres. Where a probabilistic atlas was used to define the ROI, the classification threshold is given (i.e. only voxels with a probabilistic value above this threshold were included).

ROI label	Description
A1	Primary auditory cortex, based on areas TE 1.0, 1.1, and 1.2 in the Jülich histological (cyto- and myelo-architectonic) atlas (threshold > 50%).
V1	Primary visual cortex, based on area 17/V1 in the Jülich histological (cyto- and myelo-architectonic) atlas (threshold > 50%).
OPA	Sphere of 7.5-mm radius at approximate location of the occipital place area (OPA), based on average MNI coordinates (left: −29.4, −83.8, 23.9, right: 35.7, −78.5, 23.7) provided by Sun et al. (2021). These coordinates were acquired using a scene > objects localizer, averaged across 17 participants. This ROI was included because, in a previously reported analysis (Norman and Thaler 2023), we found that expert echolocators showed greater activity in this area when listening to route sounds relative to scrambled sounds. This is consistent with the OPA’s role in boundary-based visual navigation in the sighted brain (Julian et al. 2016; Kamps et al. 2016).

#### Whole brain analysis

In addition to the ROI analysis, we also ran a whole-brain analysis to show the difference in activation between the post- and pretraining sessions for each contrast (same as those used in the ROI analysis). In order to objectively assign anatomical labels to activation clusters from the whole brain analysis, the coordinates of the peak activity within each cluster were extracted, along with the coordinates of the local maxima within each cluster, and these was used to extract corresponding labels from the Jülich Histological Cyto-Architectonic Atlas (Eickhoff et al. 2007) and MNI structural atlas (Collins et al. 1995; Mazziotta et al. 2001). Where the atlases returned probabilistic values of at least 25% for a particular anatomical label, this label was then assigned to that cluster.

### Voxel-based morphometry

We used a longitudinal VBM analysis (Ashburner and Friston 2000) to quantify training-induced changes in gray matter density in our BPs and SPs, looking specifically at primary sensory areas (V1 and A1) and OPA with an ROI analysis and on a more general level in a whole-brain analysis. VBM analysis involves spatially normalizing the T1 images to a common template and then segmenting the images into different tissue classes, followed by smoothing. Additionally, for longitudinal VBM analysis, a mean transformation for each timepoint is additionally applied to all structural images. Voxel-wise statistical tests can then be performed on the smoothed images to assess differences in gray matter density in regions of interest or across the whole brain.

For our VBM analysis, all T1 images were first manually aligned so that the anterior commissure was set as the point of origin. We then analyzed the data using the longitudinal pipeline provided in the Computational Anatomy Toolbox (CAT12, Gaser et al. 2022; http://www.neuro.uni-jena.de/cat) for SPM12 (MATLAB 2022a, The Mathworks, Natick, MA, USA). There are two processing pipelines available for longitudinal analysis in CAT12: one optimized to detect smaller changes (e.g. plasticity or learning) and one optimized to detect larger changes (e.g. atrophy or disease). We used the first option, using the default SPM12 tissue probability maps for segmentation, the DARTEL IXI555 MNI152 template for spatial registration (1.5 mm voxel size, Ashburner 2007), segmentation of gray and white matter (Ashburner and Friston 2005), and spatial smoothing of 8 mm (the CAT12-recommended level). The “Estimate TIV” tool in CAT12 was also used to estimate total intracranial volume for each subject, to be used as a covariate in the analysis. The same ROIs used in the functional analysis were used here to extract the regional estimates of gray matter density.

## Results

### Data availability

Processed participant data (age, group, behavior, ROI results) are available as Supplemental Material S1.

### Behavioral performance in the training program

Participants’ improvement in behavioral performance in the training program have been reported in detail previously (Norman et al. 2021), and relevant results are also provided in the Supplemental Material S2 and Supplemental Figs S1–S5. To summarize briefly here, both BP and SP groups showed clear improvements in echolocation ability across all training tasks (size discrimination, orientation perception, and a virtual echo-acoustic maze navigation). For example, for the virtual echo-acoustic maze navigation task, the mean time taken to navigate the virtual mazes fell from 104.1 s to 40.9 s in SPs and from 137.0 s to 57.23 s in BPs. For the orientation perception task, the proportion of correct responses rose from 40.2% to 75.6% in SPs and 36.1% to 62.3% in BPs (chance performance was 25%). For the size discrimination task, the proportion of correct responses rose from 55.5% to 83.6% SPs and 53.3% to 74.0% in BPs (chance performance was 50%). Please see Supplemental Material S2 for more details.

### Behavioral performance in the experimental task (pre- and post-training)

**Fig. 5:**
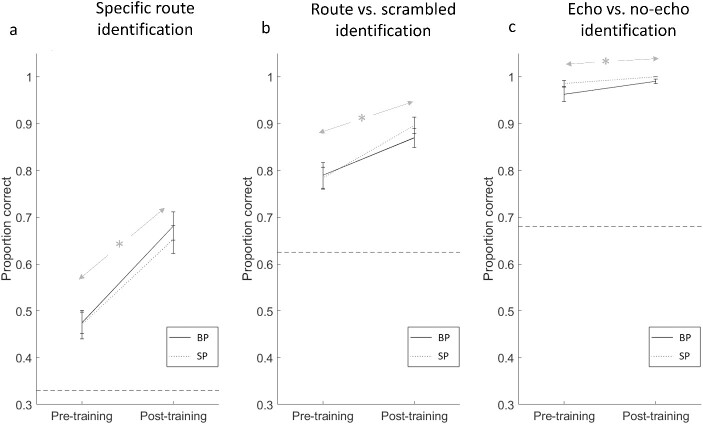
Data from the behavioral task, showing improvement in performance following training for BPs and SPs. Three separate measures of performance are given: ability of participants to identify specific route types a), to identify coherent route sounds vs. scrambled sounds b), and to identify the sounds containing echoes from those that do not (c). The asterisk indicates a significant training effect (with no interaction with group). Horizontal dashed lines show chance performance. Error bars show standard error of the mean.

For the computer-based echolocation task collected prior to fMRI scanning, we calculated the proportion of correct responses separately at pre- and post-training for three different measures of performance: specific route identification, route vs. scrambled identification, and echo identification. Group means for these data are shown in Fig. 5. A detailed breakdown of errors for the different conditions, groups, and pre and post is provided in the Supplemental Material S2. A mixed ANOVA (with subject group as the between-subject variable and timepoint as the within-subject variable) was used to test for effects of subject group and training. Behavioral performance during fMRI was also analyzed in the same way, and the pattern of results was consistent with what we observed prior to scanning. We found the in-scanner measure to be more variable, however, due to participants pressing more than one key accidentally or failing to respond on some trials. For SPs, this happened on average on 0.69% of trials in the pretraining session and 0.13% in the post-training session. In BPs, this happened on average on 2.04% of trials in the pretraining session and 0.31% in the post-training session. Data from these trials were still included in the fMRI analysis.

#### Specific route identification

When considering specific route identification, a response was correct when participants identified the specific route (single turn; two-turn-same; two-turn-different) when it was presented. Thus, specific route identification measures participants’ ability to correctly identify specific echo-acoustic routes. There was a significant effect of training [*F*(1,24) = 47.587, *P* < 0.001 η_p_^2^ = 0.665], with participants being more accurate at post-training (mean = 0.666) compared to pretraining (mean = 0.472). There was no significant difference between groups [*F*(1,24) = 0.290, *P* = 0.595] and no interaction [*F*(1,24) = 0.183, *P* = 0.672].

#### Route vs. scrambled identification

When considering scrambled vs. route identification, a response was identified as correct not only when participants gave a “scrambled” response to a scrambled sound but also when they gave any of the route responses when any of the route sounds were presented (regardless of whether it was a single turn, two-turn-same, or two-turn-different). Thus, scrambled vs. route identification measures participants’ ability to distinguish spatially coherent echo-acoustic sounds from spatially incoherent echo-acoustic sounds. There was a significant effect of training [*F*(1,24) = 45.273, *P* < 0.001 η_p_^2^ = 0.654], with participants being more accurate at post-training (mean = 0.884) compared to pretraining (mean = 0.786). There was no significant difference between groups [*F*(1,24) = 0.128, *P* = 0.724] and no interaction [*F*(1,24) = 1.258, *P* = 0.273].

#### Echo vs. no-echo identification

When considering echo identification, a response was identified as correct when participants responded with “no echo” when stimuli containing no echoes were present and also when participants gave any other response when any of the other stimuli were presented (e.g. if a “single turn” route was labeled as “scrambled,” then this would be classed as correct because the sound contains echoes). Thus, echo identification measures participants’ ability to distinguish echo from nonecho sounds. There was a significant effect of training [*F*(1,24) = 9.494, *P* = 0.005 η_p_^2^ = 0.283], with participants being more accurate at post-training (mean = 0.996) compared to pretraining (mean = 0.975). There was no significant difference between groups [*F*(1,24) = 3.108, *P* = 0.091] and no interaction [*F*(1,24) = 0.969, *P* = 0.335].

Overall, these results suggest that BPs and SPs improved their ability to perceive echo-acoustic space and to detect the presence of echoes and, importantly, there was no difference between the abilities of BPs and SPs.

#### Correlations between behavioral performance and age

In order to assess whether a participant’s age predicts improvement in behavioral performance, we calculated correlations between age and post-pre changes in each of the three behavioral measures. There was no evidence of a correlation between age and performance changes for route identification [*r*(24) = 0.054, *P* = 0.794], route vs. scrambled identification [*r*(24) = −0.021, *P* = 0.919], or echo vs. no-echo identification [*r*(24) = 0.351, *P* = 0.079].

### fMRI ROI analysis (pre- and post-training)

To test for effects of training and participant group in each ROI and for each contrast, we used mixed ANOVA, with group (BP, SP) as the between-subject variable and timepoint (pre, post) as the within-subject variable.

**Fig. 6:**
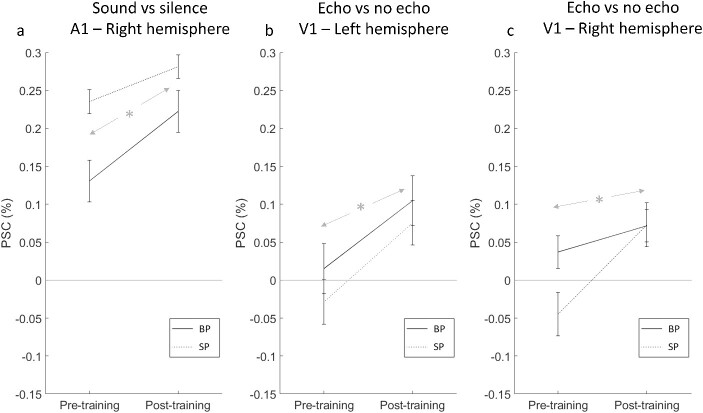
Data from the ROI analysis, showing only the results for which there was a significant effect of training. The panels show the significant effect of training in right A1 for the sound vs. silence contrast a), and the same for the echo vs. no echo contrast in left b) and right c) V1. The asterisk indicates a significant training effect (with no interaction with group). Error bars show standard error of the mean.

We found a significant training effect in right A1 for the contrast sound vs. silence [*F*(1,24) = 5.090, *P* = 0.033, η_p_^2^ = 0.175], with PSC increasing from 0.187 to 0.254. There was no difference between groups [*F*(1,24) = 1.003, *P* = 0.327] and no interaction [*F*(1,24) = 0.560, *P* = 0.461] (Fig. 6a). 8 out of 12 BPs and 8 out of 14 SPs showed a positive effect of training (i.e. post–pre difference is > 0) for sound vs. silence in right A1.

We found a significant training effect in left V1 for the contrast echo vs. no echo [*F*(1,24) = 4.948, *P* = 0.036, η_p_^2^ = 0.171], with PSC increasing from −0.009 to 0.089. There was no difference between groups [*F*(1,24) = 0.257, *P* = 0.617] and no interaction [*F*(1,24) = 0.029, *P* = 0.867] (Fig. 6b). 7 out of 12 BPs and 8 out of 14 SPs showed a positive effect of training (i.e. post–pre difference is > 0) for echo vs. no-echo in left V1.

We found a significant training effect in right V1 for the contrast echo vs. no echo [*F*(1,24) = 4.284, *P* = 0.050 (is actually 0.04977), η_p_^2^ = 0.151], with PSC increasing from −0.007 to 0.072. There was no difference between groups [*F*(1,24) = 0.450, *P* = 0.509] and no interaction [*F*(1,24) = 1.270, *P* = 0.271] (Fig. 6c). 7 out of 12 BPs and 9 out of 14 SPs showed a positive effect of training (i.e. post–pre difference is >0) for echo vs. no-echo in right V1.

None of the other training or interaction effects were significant in any ROI (see Supplemental Material S2 for full report). As expected from previous work (Norman and Thaler 2023), there were also some group differences (i.e. BPs had higher PSC in OPA for sound vs. silence contrast and echo vs. no echo contrast, and SPs had higher PSC for echo vs. no echo contrast in right A1), but these were unaffected by training (see Supplemental Material S2 for full report).

#### fMRI ROI training effects, age, and behavioral performance

We did not find any evidence of a correlation between training-related changes in PSC in any ROI and age (see Supplemental Material S2 for full report). We also did not find any evidence of a correlation between training-related changes in any ROI and training-related improvement on the behavioral tasks (see Supplemental Material S2 for full report).

#### ROI responses to no-echo and echo conditions

Furthermore, in order to determine the nature of the effect(s) underlying the training-related changes in right A1 and both left and right V1, we analyzed the PSC relative to silent baseline in these areas separately for pre- and post-training sessions and in response to no-echo and echo stimuli (i.e. scrambled and route stimuli combined) using one-sample *t* tests. Since the main analysis had not revealed differences between BPs and SPs, we considered the two groups together for these *t*-tests.

We found that in A1, as might be expected, there was a significant positive response to any sound stimuli both before [no-echo: *t*(25) = 2.911, *P* = 0.007; echo: *t*(25) = 4.967, *P* < 0.001] and after [no-echo: *t*(25) = 4.190, *P* < 0.001; echo: *t*(25) = 6.072, *P* < 0.001] training. In right V1, there was no significant positive response to any sound stimuli either before [no-echo: *t*(25) = 0.232, *P* = 0.818; echo: *t*(25) = 0.013, *P* = 0.990] or after training [no echo: *t*(25) = 0.721, *P* = 0.478; echo: *t*(25) = 1.905, *P* = 0.068], even though direct pre–post comparison had revealed a relative increase for echo stimuli (compare Fig. 6c). In left V1, we found a unique significant response specific to echo stimuli emerging after training [no echo: *t*(25) = 1.743, *P* = 0.094; echo: *t*(25) = 2.616, *P* = 0.015] that was not present before training [no echo: *t*(25) = 1.213, *P* = 0.236]; echo: [*t*(25) = 1.333, *P* = 0.194].

This demonstrates a unique significant response to echo sound in left V1 post-training, consistent with which the repeated measures analysis had highlighted an increase in sensitivity to echo sound from pre- to post training in left V1 (Fig. 6b).

### fMRI—whole-brain analysis

In order to determine effects of training on activity outside our predetermined ROIs, we also ran a whole-brain analysis. A fixed-effects analysis was first run for each subject to calculate three post-v-pre contrast maps for each stimulus contrast of interest (i.e. sound vs. silence, echo vs. no echo, route vs. scrambled). These maps were then entered into a mixed-effects analysis to test for inter-group differences. The only significant difference between groups was found for the contrast echo vs. no echo, in which SPs showed a greater effect of training in left primary motor cortex and superior parietal lobule (one joint cluster, MNI coords = −30, −58, 70; *Z* = 3.75; number voxels = 4,460). Since there were no other group differences, we investigated training effects further by collapsing BPs and SPs into a single group and tested for an overall effect of training by entering post-v-pre statistical maps into a higher-level mixed effects model (using FEAT’s inbuilt “single group average” design). *Z* (Gaussianized T/F) statistic images were thresholded using cluster-based thresholding determined by *Z* > 2.3 and a cluster significance threshold of *P* = 0.05 (corrected using Gaussian Random Field theory; Worsley 2001). Separate activation maps for BPs and SPs, with and without cluster correction, are shown in Supplemental Material S2 (Figs S6–S9).

**Fig. 7:**
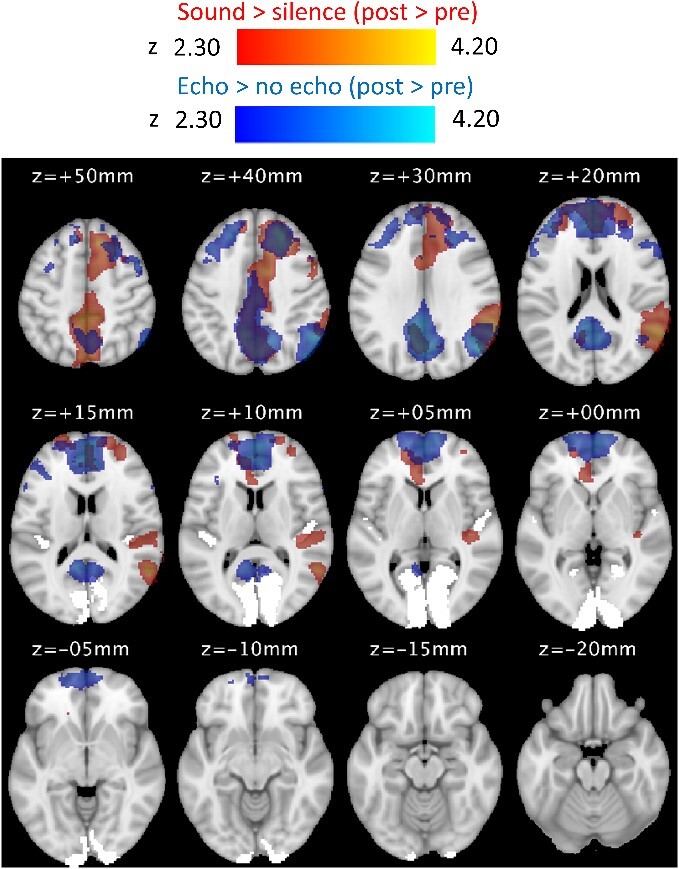
Activation maps showing the effect of training on each contrast displayed on the MNI152 standard-space template. The red maps show where activation was greater at post-training relative to pretraining for the sound vs. silence contrast (cluster-level threshold of *z* > 2.3 and *P* < 0.05). The blue maps show the same for the echo vs. no-echo contrast. No map is shown for the route vs. scrambled contrast as there were no significant clusters. BPs and SPs were entered as a single group in this analysis. Areas V1 and A1 are highlighted in white. Orientation of the images is in neurological convention (i.e. left is left).

The whole brain analysis on the effect of training for the contrasts sound vs. silence and echo vs. no-echo are shown in Fig. 7 (there were no significant training results for the route v scrambled contrast). Specifically, this shows where brain activity was greater at post-training relative to pretraining for each contrast. A detailed summary of the activation clusters for all contrasts is shown in Table 3.

**Table 3:** Summary of cluster activation peaks for the effect of training (post > pre) on each of the three stimulus contrasts. Region labels are based on MNI coordinates of the cluster peak as well as the local maxima within each cluster.

Analysis contrast	Cluster	Region label		MNI coords (mm)		*z*-stat	Num voxels
			*x*	*y*	*z*		
Sound > silence	1	GM superior parietal lobule 5 M L	2	−58	58	4.10	14,233
		GM superior parietal lobule 7A R					
		Cingulate gyrus, anterior division					
		Frontal pole					
		Supplementary motor cortex					
		Precuneous cortex					
		Superior frontal gyrus					
	2	GM inferior parietal lobule PF R	64	−54	26	4.20	2960
		GM inferior parietal lobule Pga R					
		Angular gyrus					
		Lateral occipital cortex, superior division					
		Planum temporale					
		Supramarginal gyrus, posterior division					
Echo > no-echo	1	Frontal pole	24	32	40	4.01	9115
		Middle frontal gyrus					
		Paracingulate gyrus					
		Superior frontal gyrus					
	2	GM inferior parietal lobule PGp R	52	−68	34	4.83	1884
		GM inferior parietal lobule Pga R					
		Angular gyrus					
		Lateral occipital cortex, superior division					
Route > scrambled	n/a	n/a	n/a	n/a	n/a	n/a	n/a

Abbreviation: n/a = not applicable.

For the sound vs. silence contrast, the activation maps showed a cluster centered on the superior parietal lobule (left, extending into the right), extending into the supplementary motor cortex and superior frontal gyrus. A second cluster was centered on the inferior parietal lobule (right) and extended into the lateral occipital cortex and planum temporale, extending into right A1.

For the echo vs. no-echo contrast, the activation maps showed a large cluster centered in areas of the frontal lobe (frontal pole, middle, and superior frontal gyrus). A second cluster was centered on the inferior parietal lobule in the right hemisphere and extended into the posterior divisions of V1 in both hemispheres. There were two additional clusters centered on the frontal pole (left, extending into cingulate gyrus) and inferior parietal lobule (PGp, left) respectively.

We also ran the whole brain analysis without the cluster thresholding, instead using a voxel-based thresholding of *z* > 2.3. These activation maps are reported in Supplemental Material S2 (Fig. S10).

### VBM—ROI analysis

A mixed ANCOVA, with subject group (BP, SP) as the between-subject variable, timepoint (pre, post) as the within-subject variable, and TIV (total intracranial volume) as a covariate, was used to test for effects of subject group and training in each ROI.

**Fig. 8:**
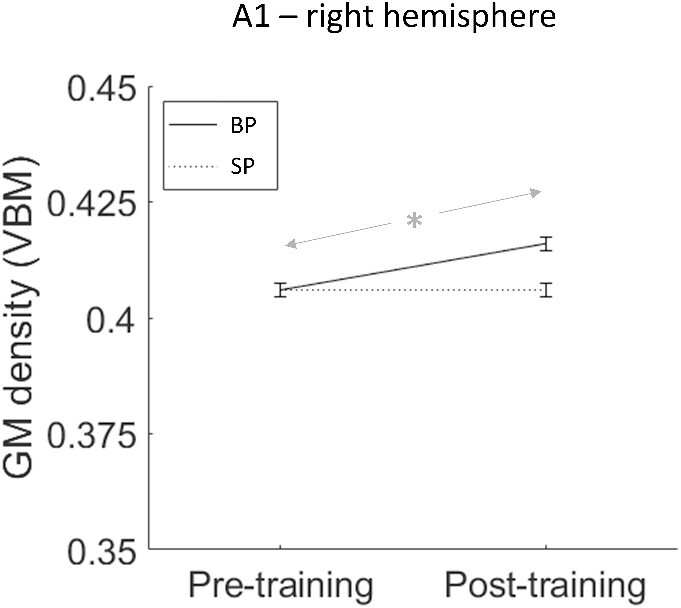
Data from the VBM ROI analysis (group means adjusted for Total Intracranial Volume, TIV), showing the only result for which there was a significant training effect. Specifically, in right A1, BPs showed a significant increase in GM density (indicated by the asterisk) but SPs did not. Error bars show standard error of the mean (with between-subject variance removed).

We found a significant training effect (Fig. 8). Specifically, right A1 showed a significant interaction effect [*F*(1,23) = 5.209, *P* = 0.032, η_p_^2^ = 0.185], and paired *t*-tests showed that this was due to BPs having higher gray matter density in right A1 post-training (adjusted mean = 0.416) compared to pretraining [adjusted mean = 0.406; *t*(11) = 3.568, *P* = 0.004], while SPs did not have a difference in gray matter density in right A1 between pre- and post-training [*t*(11) = 0.263, *P* = 0.797]. 10 out of 12 BPs and 6 out of 14 SPs showed a positive effect of training (i.e. post-pre difference is > 0) for gray matter density in right A1.

None of the other training or interaction effects were significant in any ROI (see Supplemental Material S2 for full report). There were also differences between participant groups consistent with previous literature (e.g. Ptito,, Schneider, et al. 2008; Boucard et al. 2009)—e.g. SPs having higher gray matter density in both left and right V1 as compared to BPs, but these group differences were unaffected by training (see Supplemental Material S2 for full report).

#### VBM ROI training effects, age, and behavioral performance

We did not find any evidence of a correlation between VBM training effects and age (see Supplemental Material S2 for full report). We also did not find any evidence of a correlation between VBM training effects and training-related improvement on the behavioral tasks (see Supplemental Material S2 for full report).

### VBM—whole-brain analysis

**Fig. 9:**
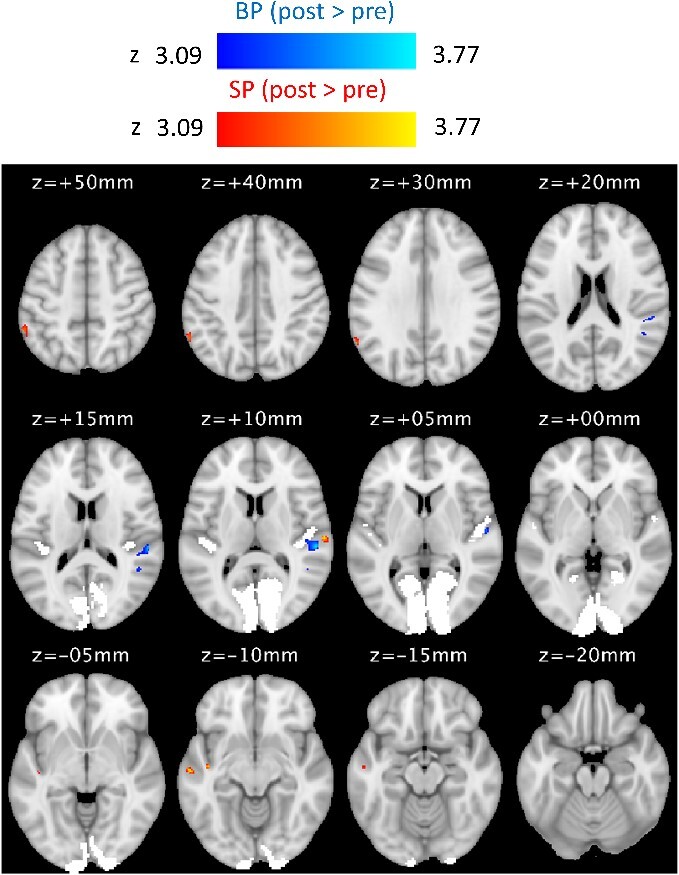
Statistical maps showing longitudinal increases in gray matter density in BPs (blue colormap) and SPs (red colormap) following 10 weeks of echolocation training (thresholded at *P* < 0.001 and cluster extent of *k* > 20). Areas V1 and A1 are highlighted in white. Orientation of the images is in neurological convention (i.e. left is left).

The whole-brain VBM analysis was run using SPM12’s contrast manager with a mixed ANOVA factorial design (statistics maps were thresholded at *P* < 0.001 with a cluster extent threshold of *k* > 20). A test for an interaction between subject group and training revealed several differences in gray matter density across the brain, which was followed by two separate paired *t* tests to examine post v pre increases in gray matter density in BPs and SPs, respectively. Fig. 9 shows the results of both *t* tests, with a detailed summary of cluster activations provided in Table 4.

**Table 4:** Summary of peak clusters identified in the VBM analysis where BPs and SPs showed higher gray matter density post-training relative to pretraining.

Subject group	Cluster	Region label		MNI coords (mm)		*z*-stat	Num voxels
			*x*	*y*	*z*		
BP	1	GM primary auditory cortex TE1.0 R	57	−26	14	3.72	232
		GM primary auditory cortex TE1.1 R					
	2	GM primary auditory cortex TE1.0 R	57	−12	5	3.41	22
	3	GM inferior parietal lobule Pga R	47	−48	18	3.36	34
SP	1	Planum temporale	63	−18	9	3.77	63
	2	Angular gyrus	−57	−51	45	3.66	213
		Supramarginal gyrus, posterior division					
	3	Middle temporal gyrus, posterior division	−59	−17	−11	3.64	45
	4	Planum polare	−42	−12	−11	3.57	26
	5	Inferior temporal gyrus, posterior division	57	−18	−36	3.50	25
	6	Temporal fusiform cortex, posterior division	−26	−38	−23	3.31	24

BPs showed higher gray matter density post-training in right primary auditory cortex and right inferior parietal lobule. SPs showed higher gray matter density post-training in right temporal areas (planum temporale, inferior temporal gyrus), left temporal areas (planum polare, middle temporal, and fusiform gyrus), and inferior parietal cortex (the angular gyrus). Notably, the cluster in right temporal lobe where SPs show training related gray matter increase is adjacent to the cluster where BPs show training related gray matter increase (compare slice at *z* = +10 mm in Fig. 9).

## Discussion

We show here, for the first time, functional and structural brain changes in primary sensory areas V1 and A1 in blind and sighted people who learn click-based echolocation in adulthood. These results are a key finding with respect to previous studies that found plasticity in blind and sighted adult people primarily in higher-order sensory areas (e.g. Amedi et al. 2007; Ptito et al. 2009; Reich et al. 2011; Striem-Amit et al. 2012; Siuda-Krzywicka et al. 2016; Aggius-Vella et al. 2023).

Our experimental task was based on virtual echo-acoustic navigation and allowed us to measure changes in brain activation in response to three different levels of stimulus processing: (i) sound per se, (ii) echoes per se, and (iii) spatiotemporal echo-acoustic information. Although we did not find any evidence of training-induced changes related to spatiotemporal information, we did find, using an ROI approach, increased brain activation for perceiving echoes per se in left and right V1 in our BPs and SPs. It is now established that V1 is recruited for echo-acoustic processing in blind echolocation experts (10 years or more of daily use) (e.g. Thaler et al. 2011; Wallmeier et al. 2015; Flanagin et al. 2017; Norman and Thaler 2019; Norman and Thaler 2023). Our results are generally consistent with that and demonstrate functional plasticity associated with comparably short-term 10 weeks of echolocation learning in both BPs and SPs. This provides strong evidence that the ability of a primary sensory area (V1) to exhibit sensitivity to input from a different modality (here: sound echoes) can be considered a normal characteristic of the typical adult human brain.

Further to this, we found training-induced changes in functional activity related to sound per se in right A1 in BPs and SPs, as well as an increase in gray matter density in right A1 in BPs and in adjacent areas (i.e. planum temporale and inferior temporal gyrus) in SPs. The functions of left and right A1 are considered relatively distinct in the human brain, with right A1 being thought of as specialized for spectral processing and left A1 thought of as specialized for temporal processing (Zatorre et al. 2002). Spectral information is considered an especially important cue for echo detection and discrimination in humans (Schenkman and Nilsson 2011; Norman and Thaler 2020, 2021). It is possible that the observed training-related increase in activity in right A1 reflects an improved ability to process the spectrum of sounds in our stimuli (i.e. clicks alone and clicks with echoes). The increase in the VBM signal indicates that a greater concentration of gray matter was present in right A1 at post-training compared to pretraining. While macroscopic variations in brain *structure* (e.g. gray matter density) are known to be associated with behavioral or perceptual performance (see Bermudez and Zatorre 2005; Ditye et al. 2013; Kanai and Rees 2011; Kwok et al 2011; Yoshimura et al. 2017; Boucard et al. 2009), it is currently unclear what specific cellular changes drive gray matter changes as detected with VBM. In nonhuman animals at least, learning-induced structural changes detected by VBM are more likely to reflect increases in dendritic spine density over other changes such as increases in nuclei density or neuronal size, and this does not necessarily covary with cortical thickness (Keifer Jr et al. 2015).

In our whole-brain analysis, we also found evidence of additional training-induced increases in functional activation in other brain areas beyond V1 and A1. For changes in general acoustic processing, a cluster centered on the left and right superior parietal lobules was observed, extending into frontal cortex. A second cluster was also observed centered on the inferior parietal lobules. The most likely explanation for this is a training-related increase in attention to the stimuli, given that such parietal areas are considered to be part of the dorsal frontoparietal attention network (Szczepanski et al. 2013)—a network that is thought to control top–down attention to environmental objects and tasks (Corbetta and Shulman 2002;Corbetta et al. 2008; Shomstein and Yantis 2006).

For functional changes associated with echo perception per se, one cluster was observed in the frontal lobe (frontal pole, middle, and superior frontal gyrus) and paracingulate gyrus. It is possible that this increase in activation in frontal areas represents cognitive processing related to task goals and behavior monitoring (e.g. Sakai 2008). There was also a second cluster centered on the inferior parietal lobule (right), which extended into the lateral occipital cortex and anterior V1, thus corroborating our ROI analysis. This cluster also overlapped well with the precuneus (bilaterally)—an area that is involved in a wide range of integrative tasks (see Cavanna and Trimble 2006), including memory-dependent spatial navigation (Brodt et al. 2016). This echo vs. no echo cluster also covers significant portions of the retrosplenial cortex (Brodmann’s areas 29 and 30, bilaterally)—a crucial part of the spatial navigation network (Vann et al. 2009). Although the echo vs. no-echo contrast does not selectively target changes in navigation-related activation (unlike the route vs. scrambled contrast), its patterns of activation might nonetheless indicate that these areas typically involved in spatial navigation become more active with training in response to auditory-spatial stimuli generally compared to nonspatial stimuli.

Functional changes delineated by our whole-brain fMRI analyses appear to overlap nodes of the default mode network (DMN). Since its discovery, there has been considerable research into the DMN, its anatomical substrates and functions (e.g. for review, see Smallwood et al. 2021). To visualize overlap between our fMRI results and DMN nodes, we superimposed a DMN mask (Wang et al. 2020) onto our results (after applying smoothing and binarization) [see Supplemental MaterialsS2 (Fig. S11)].

Our whole-brain analysis of the VBM data also revealed additional areas of increased gray matter density that were specific to BPs (right inferior parietal lobule) and SPs (planum polare, middle temporal gyrus, fusiform gyrus, and angular gyrus). It’s possible that these structural changes relate to additional increases in task-related attention and acoustic stimulus processing.

Although our BPs and SPs showed significant perceptual improvements and training-induced functional and structural plasticity, they do not show training induced changes in OPA for spatial echo processing for navigation. In previous work (Norman and Thaler 2023), we had used the same fMRI protocol to reveal activity in OPA and other areas of navigation networks for echo-acoustic navigation in blind expert echolocators (as opposed to blind and sighted controls). We had also found correlations between task performance and task-relevant recruitment of OPA in EEs (correlation between route vs. scrambled accuracy and OPA recruitment for route vs. scrambled). The expert echolocators in our previous study had 10 years or more of daily echolocation use, thus vastly exceeding 10 weeks of training, which we investigated here. As such, the lack of OPA recruitment (and lack of correlation) as observed in our current study might indicate that additional training and/or experience with echolocation is required for functional recruitment of OPA for echo-acoustic navigation. We do not think that our results are inconsistent with previous work showing that even much shorter periods of training with, for example, vision-to-touch sensory substitution are associated with brain activity in visual areas in a spatial navigation task in congenitally blind subjects (e.g. Kupers et al. 2010). From a methodological point of view, there are various differences between the studies in terms of task and scanning, and the fact that our study used a pre vs. post comparison, while Kupers et al. (2010) looked at brain activity only after training. Further, and importantly, our fMRI paradigm was sensitive enough to detect changes for other aspects of performance, e.g. increase in activity in V1 for the contrast “echo vs. no-echo,” even in sighted people.

In our analysis of training-induced changes in functional activity, we found that BP and SP groups were largely similar to one another—that is, there was only small evidence that any training effects were dependent on subject group. There were some group differences in training-related changes in gray matter density, however. Thus, while the functional data might support the idea that neuroplastic change is fundamentally constrained by an underlying structural “blueprint” common to all brains (Makin and Krakauer 2023), the VBM data suggest that a more nuanced view may be required. For example, while similar increases in activity in V1 and A1 have been observed, this does not necessarily mean that the same mechanisms took place in the two groups. Further research is needed to explore this, but the current study nonetheless provides evidence that long-term vision loss is not a necessary precondition for functional plasticity-related changes in early visual cortex (see also Merabet et al. 2008; Siuda-Krzywicka et al. 2016). This could have implications for the successful rehabilitation of people with progressively degrading visual conditions.

In our sample of blind participants, 11 had vision loss present from birth and 1 from 3 months of age. Even though blindness was profound for all blind participants at time of testing, nearly all of them were exposed to at least some level of vision during critical development years in childhood (even if it was severely limited, like the ability to sense bright light). Thus, our participants are different from participants who are congenitally totally blind. Any change we observe here in our study has taken place in adulthood, i.e. well after the critical period for vision has closed. Considering how early in life and how profound vision loss was for our blind participants, the similarity of their results compared to those in our typically sighted participants strongly suggests that any variations in the residual visual experience of our blind participants play a limited role for the effects we observed. Yet, further research is needed with congenitally totally blind participants to investigate the potential role of visual input at/shortly after birth on adult plasticity as observed here.

One may wonder based on our findings what function V1 might serve for echolocation. While the lack of change in response to sound per se (i.e. sound vs. silence contrast) rules out a strictly cross-modal response, it is nevertheless clear that the information provided by sound echoes, i.e. input from a different modality, drives V1 in our BPs and SPs. Possibilities are that the echo-related activity increase could be due to processes to do with stimulus predictability (Rao and Ballard 1999) or spatiotemporal sequence learning (Xu et al. 2012). In our paradigm, however, these explanations would not predict a selective preference for echoes, i.e. they would predict V1 to be equally active for stimuli containing echoes and stimuli not containing echoes, as the timing and sequencing of the sounds in each condition were matched. Thus, the pattern of activity as well as the change in response to training we found in V1 is not a good fit for this interpretation. An alternative, and perhaps more plausible, explanation is that V1 contributes to some sort of spatial computation. In this case, one would expect V1 to be more active in the presence than in the absence of echoes, because in our paradigm the trains of sounds with echoes contain more spatial information than those without echoes. The observation that blind expert echolocators exhibit an ordered mapping of sound location in V1 (for echo as well as non-echo spatial sound) similar to sighted retinotopy in V1 (Norman and Thaler 2019) is consistent with this idea. We are not the first to propose that visual cortex could potentially serve “supra-modal” spatial functions (Pascual-Leone and Hamilton 2001; Amedi et al. 2017). A similar supra-modal spatial function has also been suggested for certain parts of auditory cortex (Lomber et al. 2010).

In conclusion, our results are difficult to reconcile with the view that sensory cortex is strictly organized by modality, as both sighted and blind participants showed increased echo-acoustic related activity in V1 in response to training. The functional similarity we observed between SPs and BPs is generally consistent with the idea that neuroplastic change may be fundamentally constrained by an underlying structural “blueprint” common to all brains (Makin and Krakauer 2023). Yet, in particular, our analyses on gray matter density also showed differences between the groups suggesting that a more nuanced view may be required. While previous studies have also addressed these important issues, we here provide evidence from the earliest cortical sensory areas, which are central to discussions around brain plasticity and organization.

## Supplementary Material

supplemental_materials_bhae239

supplemental_datasets_bhae239
