# Supplementary material for: Changes in primary visual and auditory cortex of blind and sighted adults following 10 weeks of click-based echolocation training
Source: Cereb Cortex. 2024 Jun 20;34(6):bhae239. doi: 10.1093/cercor/bhae239 (PMC11186672; doi:10.1093/cercor/bhae239)
Supplement: supplemental_materials_bhae239 [file supplemental_materials_bhae239.docx]

**Changes in primary visual and auditory cortex of blind and sighted adults following 10 weeks of click-based echolocation training.**

**Supplemental materials**

Authors: Liam J. Norman^1^, Tom Hartley^2^ and Lore Thaler^1^

*^1^Department of Psychology, Durham University, Durham, DH1 3LE*

*^2^Department of Psychology, University of York, Heslington, YO10 5DD*

*Correspondence:*

*Lore Thaler (*[*lore.thaler@durham.ac.uk*](mailto:lore.thaler@durham.ac.uk)*)*

**Table of Contents**

[Behavioural performance in the training program 4](#_Toc166572196)

[Size discrimination performance (Figure S1) 4](#_Toc166572197)

[Orientation discrimination performance (Figure S2) 5](#_Toc166572198)

[Maze completion time (Figure S3) 6](#_Toc166572199)

[Maze number of collisions (Figure S4) 7](#_Toc166572200)

[Maze proportion of successful completions (Figure S5) 9](#_Toc166572201)

[Responses in the experimental task for the different conditions, groups and pre and post 11](#_Toc166572202)

[**Table S1** Sighted Participants’ average response frequencies 11](#_Toc166572203)

[**Table S2** Blind Participants’ average response frequencies 12](#_Toc166572204)

[fMRI ROI analysis – full report 13](#_Toc166572205)

[Sound vs silence 13](#_Toc166572206)

[A1L 13](#_Toc166572207)

[A1R 13](#_Toc166572208)

[V1L 14](#_Toc166572209)

[V1R 15](#_Toc166572210)

[OPAL 15](#_Toc166572211)

[OPAR 16](#_Toc166572212)

[Echo vs no echo 16](#_Toc166572213)

[A1L 16](#_Toc166572214)

[A1R 17](#_Toc166572215)

[V1L 17](#_Toc166572216)

[V1R 18](#_Toc166572217)

[OPAL 18](#_Toc166572218)

[OPAR 19](#_Toc166572219)

[Route vs scrambled 20](#_Toc166572220)

[A1L 20](#_Toc166572221)

[A1R 20](#_Toc166572222)

[V1L 21](#_Toc166572223)

[V1R 21](#_Toc166572224)

[OPAL 22](#_Toc166572225)

[OPAR 22](#_Toc166572226)

[VBM ROI analysis – full report 23](#_Toc166572227)

[A1_L 23](#_Toc166572228)

[A1_R 24](#_Toc166572229)

[V1_L 24](#_Toc166572230)

[V1_R 25](#_Toc166572231)

[OPA_L 25](#_Toc166572232)

[OPA_R 26](#_Toc166572233)

[Additional supplemental figures S6 – S11 27](#_Toc166572234)

[References 33](#_Toc166572235)

# Behavioural performance in the training program

Participants’ improvement in behavioural performance in the training program have been reported in detail previously (Norman *et al*, 2021). Please see below for relevant results in the virtual navigation task, the orientation perception task and the size discrimination task. Performance for echolocation in real-world context was not measured in a lab-based setting, but self-reports from participants obtained 3 months after the conclusion of the training study showed that they experienced improvements due their training in and use of echolocation in their orientation and mobility in everyday life. For more details please see Norman et al. (2021).

## Size discrimination performance (Figure S1)

Proportion correct changed significantly across sessions (F_GG_ (6.831, 163.955) = 9.312; p < .001; η^2^_p_: .280), and a significant linear trend is consistent with the idea that performance improved as training progressed (F(1,24) = 45.449; p < .001; η^2^_p_: .654). On average, proportion correct improved from .54 (session 1) to .79 (session 20). This pattern is also evident in the data shown in fig S1 below. There was no difference between participant groups (F(1,24) = 2.801; p = .107, η^2^_p_: .105) or interaction between group and session (F_GG_ (6.831, 163.955) = 1.422; p = .201; η^2^_p_: .056)


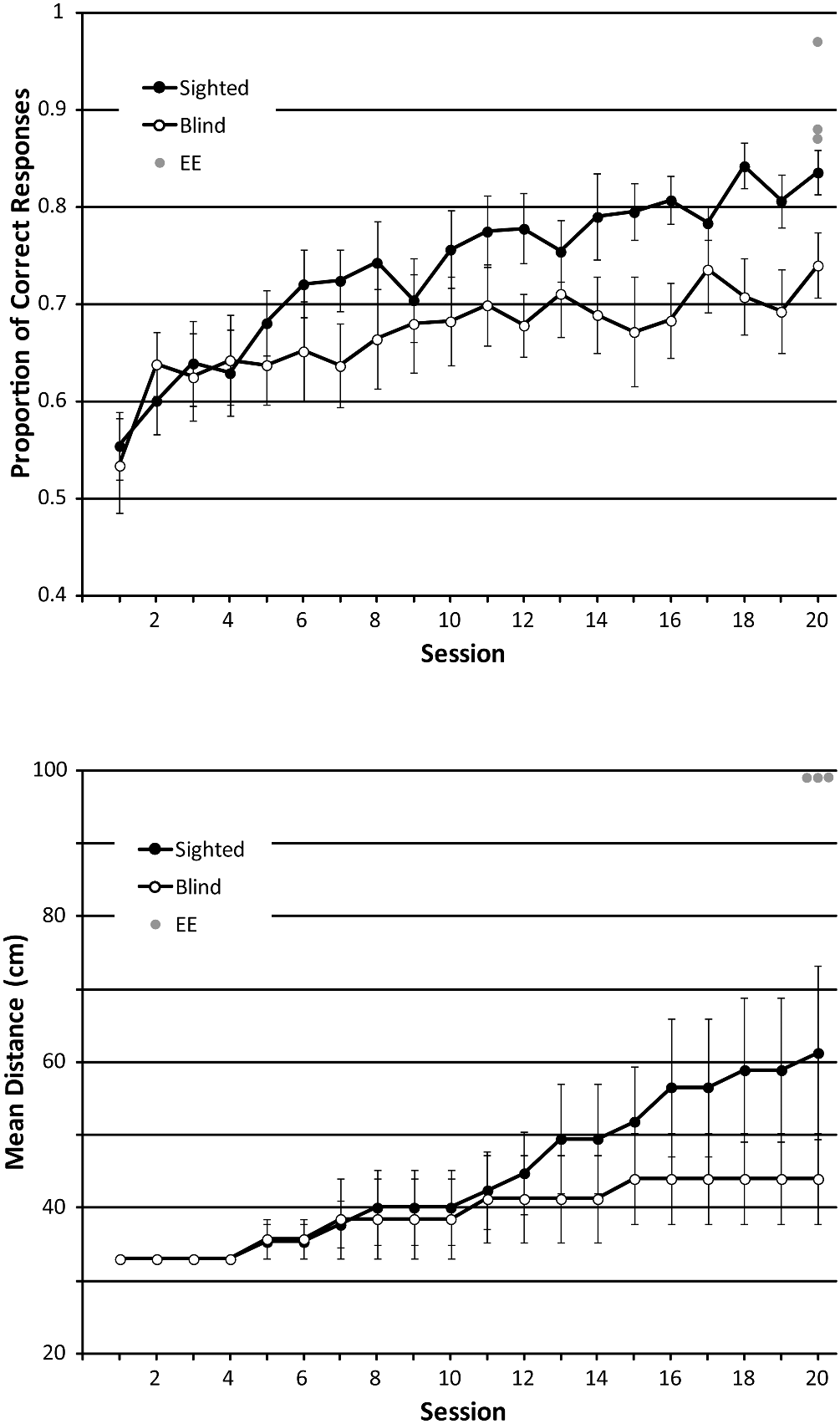


**Figure S1** Proportion of correct answers across sessions. Chance level for proportion correct is.5. Data from SPs and BPs are shown as black and white circles, respectively with each symbol representing the average and error bars representing the standard error of the mean across participants. Data from experts (n = 3) who completed only a single session without training and positioned at 100 cm distance are shown as grey circles. For comparison, they have been plotted at session 20.

## Orientation discrimination performance (Figure S2)

Proportion correct changed significantly across sessions (F_GG_ (7.007, 168.169) = 9.882; p < .001; η^2^_p_: .292), and significant linear and quadratic trends are consistent with the idea that performance improved as training progressed (linear: F(1,24) = 39.581; p < .001; η^2^_p_: .623; quadratic: F(1,24) = 6.936; p = .015; η^2^_p_: .224). On average, proportion correct improved from .38 (session 1) to .69 (session 20). This pattern is also evident in the data shown in figure S2 below. Based on the overall ANOVA, there was no difference between groups (F(1,24) = 2.967; p = .098, η^2^_p_: .110) or interaction between group and session (F_GG_ (7.007, 168.169) = 1.086; p = .374; η^2^_p_: .043).


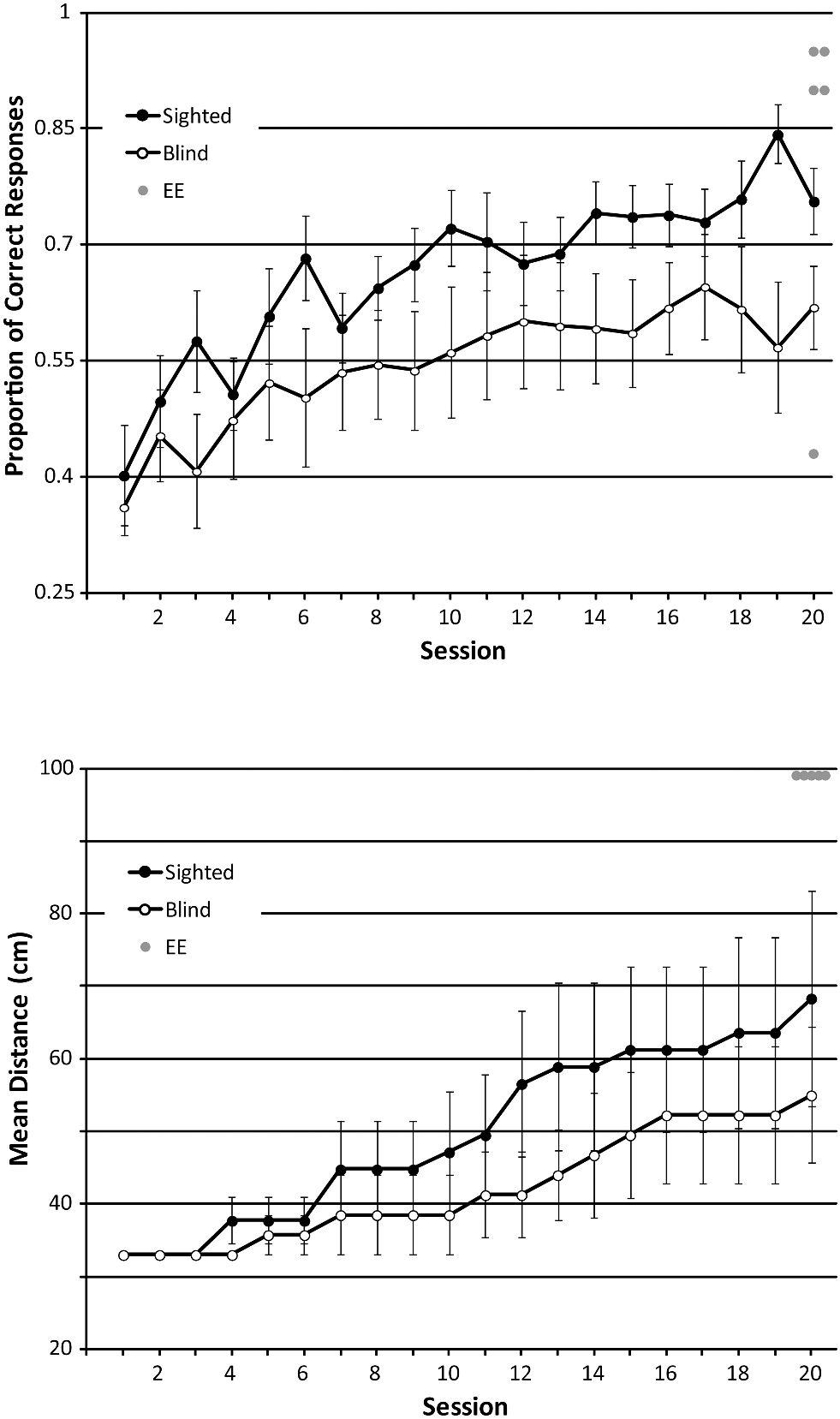


**Figure S2** Proportion of correct answers across sessions. Chance level for proportion correct is .25. Data from SPs and BPs are shown as black and white circles, respectively with each symbol representing the average and error bars representing the standard error of the mean across participants. Data from experts (n = 5) who completed only a single session without training and positioned at 100 cm distance are shown as grey circles. For comparison, they have been plotted at session 20.

## Maze completion time (Figure S3)

For sessions 1 through 14, maze completion time changed significantly across sessions 1 through 14 (F_GG_(3.474, 83.376) = 46.779; p < .001; η^2^_p_: .661), and significant linear and quadratic trends are consistent with the idea that participants became faster as training progressed (linear: F(1,24) = 85.635; p < .001; η^2^_p_: .781; quadratic: F(1,24) = 49.237; p < .001; η^2^_p_: .672). On average, maze completion time reduced from 119.30 s (session 1) to 48.42 s (session 14). This pattern is also evident in the data shown in figure S3 below. The ANOVA showed no main effect of participant group (F(1,24) = 2.399; p = .135, η^2^_p_: .091) or interaction between group and session (F_GG_ (3.474, 83.376) = 2.233; p = .081; η^2^_p_: .085).

From session 15 through 20 (during which time, the task was made more challenging), completion time changed again significantly across sessions (F(5, 120) = 7.997, p < .001; η^2^_p_: .250), and a significant linear trend is consistent with participants becoming faster as training progressed (F(1,24) = 21.384; p < .001; η^2^_p_: .471). On average, completion time reduced from 110.21 s (session 15) to 90.63 s (session 20). Throughout these sessions, SCs were faster (mean time: 78.99 s) than BCs (mean time: 115.93 s; F(1,24) = 9.553; p = .005, η^2^_p_: .285), providing evidence that SCs were less affected by the error timeouts and random starting orientations compared to BCs. There was no significant interaction between group and session (F(5, 120) = .615; p = .689; η^2^_p_: .025).


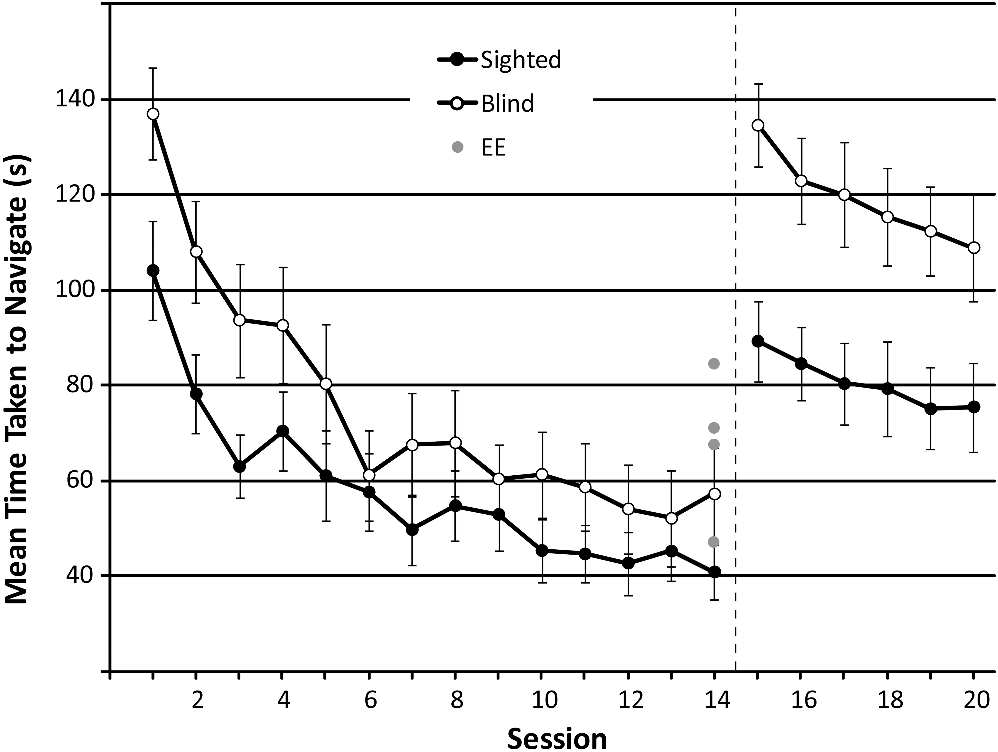


**Figure S3 The mean time taken (seconds) to complete various mazes in sessions 1–20.** In session 15, unpredictable starting orientations were introduced, along with a 15 s timeout when a collision occurred. This is represented by the dashed black line. Data from SPs and BPs are shown as black and white circles, respectively with each symbol representing the average and error bars representing the standard error of the mean across participants. Data from experts (n = 4) who completed only a single session without training are shown as grey circles. For comparison, they have been plotted at session 14.

## Maze number of collisions (Figure S4)

For sessions 1 through 14, the number of collisions changed significantly across sessions 1 through 14 (F_GG_(3.779, 90.697) = 6.753, p < .001; η^2^_p_: .220), and a significant linear trend is consistent with the idea that collisions became fewer as training progressed (F(1,24) = 29.399; p < .001; η^2^_p_: .551). On average, the number of collisions reduced from 6.57 (session 1) to 3.64 (session 14). This pattern is also evident in the data shown in figure S4. Throughout these sessions, SCs had fewer collisions (mean: 3.85; SD: 3.18) than BCs (mean: 6.95; SD: 3.18; F(1,24) = 6.138; p = .021, η^2^_p_: .204). The interaction between group and session was not significant (F_GG_ (3.779, 90.697) = .761; p = .547; η^2^_p_: .031).

For sessions 15 through 20 (during which time, the task was made more challenging), minimum effect size for the main effect of ‘session’ and the interaction effect was .100, and for the main effect of ‘group’ it was .247. The number of collisions changed again significantly across sessions (F(5, 120) = 5.659, p < .001; η^2^_p_: .191), and significant linear and quadratic trends are consistent with the idea that collisions became fewer as training progressed (linear: F(1,24) = 15.153; p = .001; η^2^_p_: .387; quadratic: F(1,24) = 15.290; p = .001; η^2^_p_: .389). On average, the number of collisions reduced from 2.77 (session 15) to 2.21 (session 20). Again, SCs had fewer collisions (mean: 1.650; SD: 1.298) than BCs (mean: 3.083; SD: 1.299; F(1,24) = 7.870; p = .010, η^2^_p_: .247) throughout these sessions, and there was no significant interaction between group and session (F(5, 120) = .950; p = .451; η^2^_p_ a: .038).


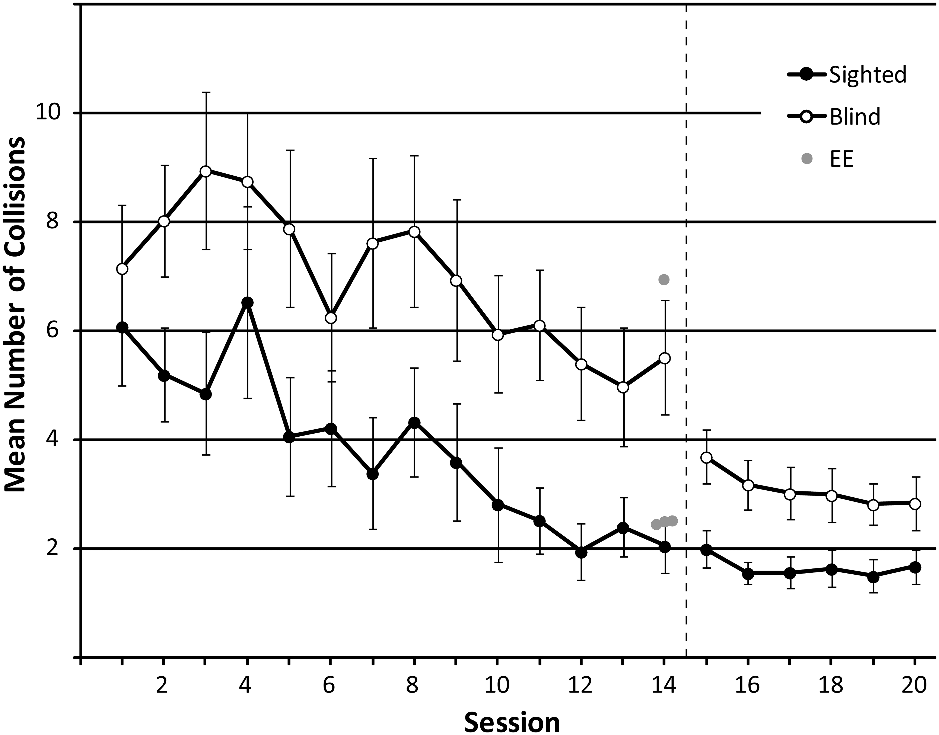


**Figure S4 The mean number of collisions made in sessions 1–20.** In session 15, unpredictable starting orientations were introduced, along with a 15 s timeout when a collision occurred. This is represented by the dashed black line. Data from SCs and BCs are shown as black and white circles, respectively with each symbol representing the average and error bars representing the standard error of the mean across participants. Data from experts (n = 4) who completed only a single session without training are shown as grey circles. For comparison, they have been plotted at session 14.

## Maze proportion of successful completions (Figure S5)

For sessions 1 through 14, the proportion of mazes successfully completed changed significantly across sessions (F_GG_(3.889, 93.328) = 16.303, p < .001; η^2^_p_: .405), and significant linear and quadratic trends are consistent with the idea that people successfully completed a greater proportion of mazes as training progressed (linear: F(1,24) = 33.494; p < .001; eta: .583; quadratic: F(1,24) = 24.199;p < .001; eta: .502). On average, the proportion of successfully completed mazes increased from.62 (session 1) to.94 (session 14). This pattern is also evident in the data shown in figure S5. There was no difference between participant groups (F(1,24) = 2.548; p = .124, η^2^_p_: .096) or interaction between group and session (F_GG_ (3.889, 93.328) = 2.239; p = .096; η^2^_p_: .085).

For sessions 15 through 20, minimum effect size for the main effect of ‘session’ and the interaction effect was .100, and for the main effect of ‘group’ it was .247. The proportion of mazes successfully completed changed again significantly across sessions (F(5, 120) = 4.382, p = .001; η^2^_p_: .154), and a significant linear trend is consistent with the idea that people successfully completed a greater proportion of mazes as training progressed (F(1,24) = 16.640; p < .001; eta: .409). On average, the proportion of successfully completed mazes increased from .72 (session 15) to .81 (session 20). Again, SCs completed a higher proportion of mazes successfully (mean: .88) compared to BCs (mean: .66) throughout these sessions (F(1,24) = 7.612; p = .011, η^2^_p_: .241), with no significant interaction between group and session (F (5, 120) = 2.093; p = .071; η^2^_p_: .08).


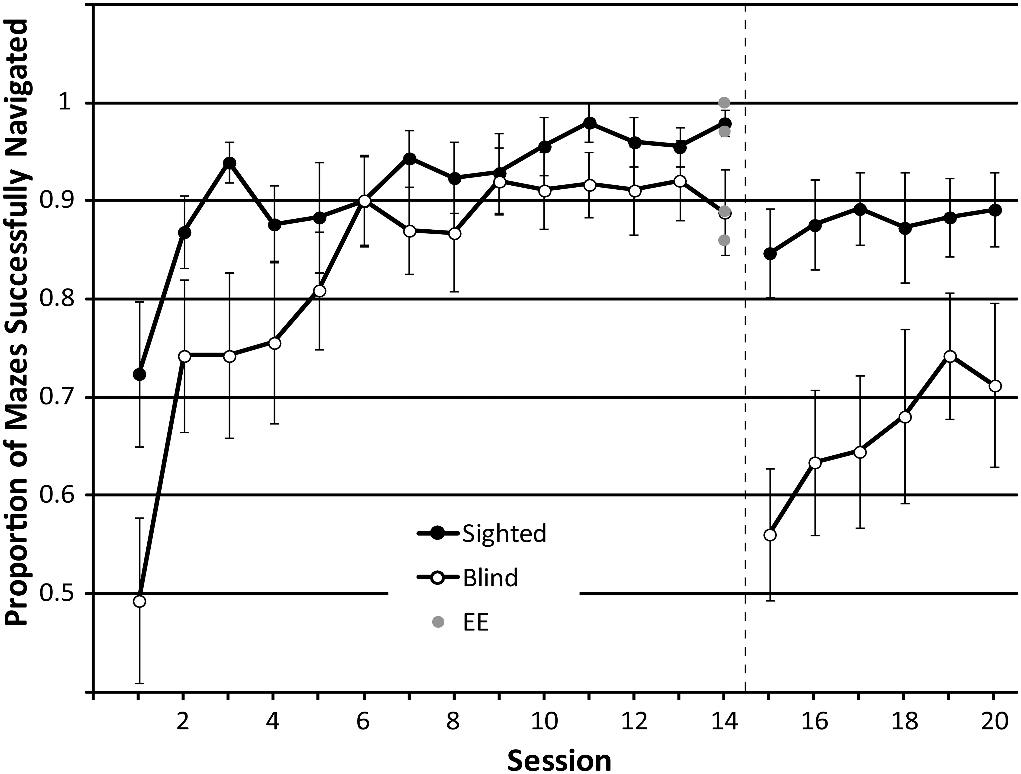


**Figure S5 The average proportion of successful maze completions in sessions 1–20.** In session 15, unpredictable starting orientations were introduced, along with a 15 s timeout when a collision occurred. This is represented by the dashed black line. Data from SCs and BCs are shown as black and white circles respectively, with each symbol representing the average and error bars representing the standard error of the mean across participants. Data from experts (n = 4) who completed only a single session without training are shown as grey circles. For comparison, they have been plotted at session 14.

# Responses in the experimental task for the different conditions, groups and pre and post

## **Table S1** Sighted Participants’ average response frequencies

|  |  |  |  |  |  |  |
| --- | --- | --- | --- | --- | --- | --- |
| **Pre** |  |  |  |  |  |  |
|  |  | **Stimulus** |  |  |  |  |
|  |  | Single turn | Two same | Two different | Scrambled | No echo |
| **Response** | Single turn | 8.07 | 3.36 | 4.36 | 2.36 | 0.21 |
|  | Two same | 2.07 | 3.86 | 2.00 | 2.43 | 0.07 |
|  | Two different | 1.57 | 3.71 | 3.36 | 2.29 | 0.00 |
|  | Scrambled | 0.29 | 0.86 | 2.00 | 4.86 | 0.00 |
|  | No echo | 0.00 | 0.21 | 0.29 | 0.07 | 11.71 |
|  |  |  |  |  |  |  |
| **Post** |  |  |  |  |  |  |
|  |  | **Stimulus** |  |  |  |  |
|  |  | Single turn | Two same | Two different | Scrambled | No echo |
| **Response** | Single turn | 10.93 | 1.64 | 3.86 | 1.07 | 0.00 |
|  | Two same | 0.36 | 6.64 | 2.50 | 1.71 | 0.00 |
|  | Two different | 0.50 | 3.36 | 5.36 | 1.36 | 0.00 |
|  | Scrambled | 0.21 | 0.36 | 0.29 | 7.86 | 0.00 |
|  | No echo | 0.00 | 0.00 | 0.00 | 0.00 | 12.00 |

## **Table S2** Blind Participants’ average response frequencies

|  |  |  |  |  |  |  |
| --- | --- | --- | --- | --- | --- | --- |
| **Pre** |  |  |  |  |  |  |
|  |  | **Stimulus** |  |  |  |  |
|  |  | Single turn | Two same | Two different | Scrambled | No echo |
| **Response** | Single turn | 8.42 | 3.17 | 4.42 | 2.75 | 0.25 |
|  | Two same | 1.58 | 3.08 | 2.00 | 1.33 | 0.17 |
|  | Two different | 0.75 | 4.42 | 3.25 | 1.92 | 0.17 |
|  | Scrambled | 0.92 | 1.25 | 1.50 | 5.83 | 0.25 |
|  | No echo | 0.33 | 0.08 | 0.83 | 0.17 | 11.17 |
|  |  |  |  |  |  |  |
| **Post** |  |  |  |  |  |  |
|  |  | **Stimulus** |  |  |  |  |
|  |  | Single turn | Two same | Two different | Scrambled | No echo |
| **Response** | Single turn | 10.25 | 1.58 | 3.00 | 1.17 | 0.08 |
|  | Two same | 0.50 | 7.33 | 2.42 | 1.08 | 0.00 |
|  | Two different | 0.67 | 2.33 | 5.00 | 1.42 | 0.00 |
|  | Scrambled | 0.50 | 0.50 | 1.50 | 8.25 | 0.00 |
|  | No echo | 0.08 | 0.25 | 0.08 | 0.08 | 11.92 |

# fMRI ROI analysis – full report

A mixed ANOVA (with subject group as the between-subject variable and timepoint as the within-subject variable) was used to test for effects of subject group and training in each ROI and for each contrast. Pearson’s correlations were used to test for a relationship between training-related change in PSC and change in behavioural accuracy, or age.

## Sound vs silence

### A1L

We found no significant training effect in left A1 for the contrast sound vs silence (F(1,24) = .108, p = 0.746), no difference between groups (F(1,24) = 1.979, p = 0.172), and no interaction (F(1,24) = .744, p = .397).

There was no correlation between training-related change in PSC and change in route vs. scrambled identification accuracy (r(24)=-0.067, p=.744).

There was no correlation between training-related change in PSC and change in specific route identification accuracy (r(24)=0.304, p=.131).

There was no correlation between training-related change in PSC and change in echo vs no echo identification accuracy (r(24)= -0.015, p=.943).

There was no correlation between training-related change in PSC and age (r(24)= 0.014, p=.944).

### A1R

We found a significant training effect in right A1 for the contrast sound vs silence (F(1,24) = 5.082, p = 0.034, η_p_^2^ = .175), with PSC increasing from .187 to .254. There was no difference between groups (F(1,24) = 1.004, p = 0.326, and no interaction (F(1,24) = .566, p = .459).

There was no correlation between training-related change in PSC and change in route vs. scrambled identification accuracy (r(24)=0.139, p=.497).

There was no correlation between training-related change in PSC and change in specific route identification accuracy (r(24)=0.141, p=.492).

There was no correlation between training-related change in PSC and change in echo vs no echo identification accuracy (r(24)= 0.381, p=.055).

There was no correlation between training-related change in PSC and age (r(24)= 0.209, p=.304).

### V1L

We found no significant training effect in left V1 for the contrast sound vs silence (F(1,24) = 0.287, p = 0.597, no difference between groups (F(1,24) = 1.701, p = 0.205), and no interaction (F(1,24) = 1.552, p = .225).

There was no correlation between training-related change in PSC and change in route vs. scrambled identification accuracy (r(24)=0.110, p=.592).

There was no correlation between training-related change in PSC and change in specific route identification accuracy (r(24)=-0.024, p=.908).

There was no correlation between training-related change in PSC and change in echo vs no echo identification accuracy (r(24)= 0.047, p=.818).

There was no correlation between training-related change in PSC and change in echo vs no echo identification accuracy (r(24)= 0.047, p=.821).

### V1R

We found no significant training effect in right V1 for the contrast sound vs silence (F(1,24) = 2.785, p = 0.108, no difference between groups (F(1,24) = 0.020, p = 0.890), and no interaction (F(1,24) = .552, p = .465).

There was no correlation between training-related change in PSC and change in route vs. scrambled identification accuracy (r(24)=0.193, p=.344).

There was no correlation between training-related change in PSC and change in specific route identification accuracy (r(24)=0.101, p=.623).

There was no correlation between training-related change in PSC and change in echo vs no echo identification accuracy (r(24)= 0.004, p=.986).

There was no correlation between training-related change in PSC and age (r(24)= -0.047, p=.820).

### OPAL

We found no significant training effect in left OPA for the contrast sound vs silence (F(1,24) = 0.005, p = 0.942, a significant difference between groups (F(1,24) = 11.388, p = 0.003, η_p_^2^ = .322; BPs mean = 0.155, SPs mean = -0.062), and no interaction (F(1,24) = 1.297, p = .266).

There was no correlation between training-related change in PSC and change in route vs. scrambled identification accuracy (r(24)=0.166, p=.417).

There was no correlation between training-related change in PSC and change in specific route identification accuracy (r(24)=0.032, p=.876).

There was no correlation between training-related change in PSC and change in echo vs no echo identification accuracy (r(24)= 0.167, p=.416).

There was no correlation between training-related change in PSC and age (r(24)= -0.174, p=.395).

### OPAR

We found no significant training effect in right OPA for the contrast sound vs silence (F(1,24) = 0.893, p = 0.354, no difference between groups (F(1,24) = 1.408, p = 0.247), and no interaction (F(1,24) = .151, p = .70).

There was no correlation between training-related change in PSC and change in route vs. scrambled identification accuracy (r(24)=0.042, p=.840).

There was no correlation between training-related change in PSC and change in specific route identification accuracy (r(24)=0.011, p=.957).

There was no correlation between training-related change in PSC and change in echo vs no echo identification accuracy (r(24)= 0.368, p=.065).

There was no correlation between training-related change in PSC and age (r(24)= -0.173, p=.397.

## Echo vs no echo

### A1L

We found no significant training effect in left A1 for the contrast echo vs no echo (F(1,24) = 0.003, p = 0.956), no difference between groups (F(1,24) = 1.683, p = 0.207), and no interaction (F(1,24) = 2.555, p = .123).

There was no correlation between training-related change in PSC and change in route vs. scrambled identification accuracy (r(24)=0.061, p=.767).

There was no correlation between training-related change in PSC and change in specific route identification accuracy (r(24)=-0.061, p=.765).

There was no correlation between training-related change in PSC and change in echo vs no echo identification accuracy (r(24)= -0.052, p=.800).

There was no correlation between training-related change in PSC and age (r(24)= -0.104, p=.614.

### A1R

We found no significant training effect in right A1 for the contrast echo vs no echo (F(1,24) = 0.078, p = 0.782), a significant difference between groups (F(1,24) = 8.972, p = 0.006, η_p_^2^ = 0.272; BPs mean = 0.030, SPs mean = 0.151), and no interaction (F(1,24) = 1.770, p = .196).

There was no correlation between training-related change in PSC and change in route vs. scrambled identification accuracy (r(24)=0.240, p=.237).

There was no correlation between training-related change in PSC and change in specific route identification accuracy (r(24)=-0.035, p=.864).

There was no correlation between training-related change in PSC and change in echo vs no echo identification accuracy (r(24)= -0.003, p=.988).

There was no correlation between training-related change in PSC and age (r(24)= -0.220, p=.279.

### V1L

We found a significant training effect in left V1 for the contrast echo vs no echo (F(1,24) = 4.962, p = 0.036, η_p_^2^ = .171), with PSC increasing from -0.009 to 0.089. There was no difference between groups (F(1,24) = 0.256, p = 0.617, and no interaction (F(1,24) = .029, p = .867).

There was no correlation between training-related change in PSC and change in route vs. scrambled identification accuracy (r(24)=0.183, p=.371).

There was no correlation between training-related change in PSC and change in specific route identification accuracy (r(24)=0.031, p=.881).

There was no correlation between training-related change in PSC and change in echo vs no echo identification accuracy (r(24)= 0.268, p=.186).

There was no correlation between training-related change in PSC and age (r(24)= 0.244, p=.230.

### V1R

We found a significant training effect in right V1 for the contrast echo vs no echo (F(1,24) = 4.284, p = 0.049, η_p_^2^ = .151), with PSC increasing from -0.007 to 0.073. There was no difference between groups (F(1,24) = 0.450, p = 0.509, and no interaction (F(1,24) = 1.275, p = .270).

There was no correlation between training-related change in PSC and change in route vs. scrambled identification accuracy (r(24)=0.193, p=.346).

There was no correlation between training-related change in PSC and change in specific route identification accuracy (r(24)=-0.054, p=.793).

There was no correlation between training-related change in PSC and change in echo vs no echo identification accuracy (r(24)= -0.030, p=.883).

There was no correlation between training-related change in PSC and age (r(24)= -0.008, p=.969.

### OPAL

We found no significant training effect in left OPA for the contrast echo vs no echo (F(1,24) = 0.170, p = 0.684), a significant difference between groups (F(1,24) = 12.012, p = 0.002, η_p_^2^ = 0.334; BPs mean = 0.158, SPs mean = -0.073), and no interaction (F(1,24) = 0.534, p = .472).

There was no correlation between training-related change in PSC and change in route vs. scrambled identification accuracy (r(24)=0.161, p=.432).

There was no correlation between training-related change in PSC and change in specific route identification accuracy (r(24)=-0.123, p=.549).

There was no correlation between training-related change in PSC and change in echo vs no echo identification accuracy (r(24)= -0.241, p=.236).

There was no correlation between training-related change in PSC and age (r(24)= -0.010, p=.961.

### OPAR

We found no significant training effect in right OPA for the contrast echo vs no echo (F(1,24) = 0.434, p = 0.516), no significant difference between groups (F(1,24) = 2.259, p = 0.146), and no interaction (F(1,24) = 0.298, p = .590).

There was no correlation between training-related change in PSC and change in route vs. scrambled identification accuracy (r(24)=0.013, p=.951).

There was no correlation between training-related change in PSC and change in specific route identification accuracy (r(24)=-0.047, p=.819).

There was no correlation between training-related change in PSC and change in echo vs no echo identification accuracy (r(24)= -0.175, p=.392).

There was no correlation between training-related change in PSC and age (r(24)= -0.038, p=.854.

## Route vs scrambled

### A1L

We found no significant training effect in left A1 for the contrast route vs scrambled (F(1,24) = 0.020, p = 0.888), no difference between groups (F(1,24) = 3.135, p = 0.089), and no interaction (F(1,24) = .415, p = .526).

There was no correlation between training-related change in PSC and change in route vs. scrambled identification accuracy (r(24)=0.102, p=.618).

There was no correlation between training-related change in PSC and change in specific route identification accuracy (r(24)=0.072, p=.728).

There was no correlation between training-related change in PSC and change in echo vs no echo identification accuracy (r(24)= 0.186, p=.362).

There was no correlation between training-related change in PSC and age (r(24)= 0.095, p=.645.

### A1R

We found no significant training effect in right A1 for the contrast route vs scrambled (F(1,24) = 0.382, p = 0.543), no difference between groups (F(1,24) = .019, p = 0.890), and no interaction (F(1,24) = .991, p = .329).

There was no correlation between training-related change in PSC and change in route vs. scrambled identification accuracy (r(24)=0.084, p=.685).

There was no correlation between training-related change in PSC and change in specific route identification accuracy (r(24)=0.148, p=.470).

There was no correlation between training-related change in PSC and change in echo vs no echo identification accuracy (r(24)= 0.180, p=.379).

There was no correlation between training-related change in PSC and age (r(24)= 0.267, p=.187.

### V1L

We found no significant training effect in left V1 for the contrast route vs scrambled (F(1,24) = 1.463, p = 0.238), no difference between groups (F(1,24) = 0.761, p = 0.392), and no interaction (F(1,24) = 1.198, p = .285).

There was no correlation between training-related change in PSC and change in route vs. scrambled identification accuracy (r(24)=-0.081, p=.695).

There was no correlation between training-related change in PSC and change in specific route identification accuracy (r(24)=0.334, p=.095).

There was no correlation between training-related change in PSC and change in echo vs no echo identification accuracy (r(24)= -0.349, p=.081).

There was no correlation between training-related change in PSC and age (r(24)= -0.246, p=.225.

### V1R

We found no significant training effect in right V1 for the contrast route vs scrambled (F(1,24) = 0.136, p = 0.716), no difference between groups (F(1,24) = 0.232, p = 0.635), and no interaction (F(1,24) = 0.541, p = .469).

There was no correlation between training-related change in PSC and change in route vs. scrambled identification accuracy (r(24)=-0.027, p=.895).

There was no correlation between training-related change in PSC and change in specific route identification accuracy (r(24)=0.254, p=.210).

There was no correlation between training-related change in PSC and change in echo vs no echo identification accuracy (r(24)= -0.166, p=.419).

There was no correlation between training-related change in PSC and age (r(24)= 0.002, p=.993.

### OPAL

We found no significant training effect in left OPA for the contrast route vs scrambled (F(1,24) = 0.006, p = 0.941), no difference between groups (F(1,24) = 2.696, p = 0.114), and no interaction (F(1,24) = 2.060, p = .164).

There was no correlation between training-related change in PSC and change in route vs. scrambled identification accuracy (r(24)=-0.043, p=.834).

There was no correlation between training-related change in PSC and change in specific route identification accuracy (r(24)=0.218, p=.286).

There was no correlation between training-related change in PSC and change in echo vs no echo identification accuracy (r(24)= -0.252, p=.215).

There was no correlation between training-related change in PSC and age (r(24)= 0.025, p=.902.

### OPAR

We found no significant training effect in right OPA for the contrast route vs scrambled (F(1,24) = 0.336, p = 0.568), no difference between groups (F(1,24) = 1.115, p = 0.302), and no interaction (F(1,24) = 0.023, p = .882).

There was no correlation between training-related change in PSC and change in route vs. scrambled identification accuracy (r(24)=-0.262, p=.196).

There was no correlation between training-related change in PSC and change in specific route identification accuracy (r(24)=0.265, p=.191).

There was no correlation between training-related change in PSC and change in echo vs no echo identification accuracy (r(24)= -0.281, p=.164).

There was no correlation between training-related change in PSC and age (r(24)= -0.091, p=.657.

# VBM ROI analysis – full report

A mixed ANCOVA (with subject group as the between-subject variable, timepoint as the within-subject variable, and TIV as a covariate) was used to test for effects of subject group and training in each ROI.

## A1_L

There was neither a significant effect of group (F(1,23)=.007, p=.934) nor training (F(1,23)=2.340, p=.140), and no interaction (F(1,23)=.001, p=.981).

There was no correlation between training-related change in VBM and change in route vs. scrambled identification accuracy (r(24)=-.098, p=.633).

There was no correlation between training-related change in VBM and change in specific route identification accuracy (r(24)=-.297, p=.141).

There was no correlation between training-related change in VBM and change in echo vs. no echo identification accuracy (r(24)=-.312, p=.121).

There was no correlation between training-related change in VBM and age (r(24)=.019, p=.925).

## A1_R

There was neither a significant effect of group (F(1,23)=.051, p=.824) nor training (F(1,23)=.027, p=.871), but there was a significant interaction (F(1,23)=5.209, p=.032, η_p_^2^ =.185). Paired t-tests showed that BPs had higher grey matter density post-training (adjusted mean = .416) compared to pre-training (adjusted mean = .406; t(11)=3.568, p=.004) but SPs did not (t(11)=.263, p=.797).

There was no correlation between training-related change in VBM and change in route vs. scrambled identification accuracy (r(24)=-.136, p=.508).

There was no correlation between training-related change in VBM and change in specific route identification accuracy (r(24)=-.253, p=.212).

There was no correlation between training-related change in VBM and change in echo vs. no echo identification accuracy (r(24)=-.049, p=.813).

There was no correlation between training-related change in VBM and age (r(24)=.315, p=.118).

## V1_L

There was a significant effect of group (F(1,23)=9.496, p=.005, η_p_^2^ =.292), with SPs having higher grey matter density (adjusted mean = .380) than BPs (adjusted mean = .321). There was no effect of training (F(1,23)=2.125, p=.158) and no interaction (F(1,23)=.210, p=.651).

There was no correlation between training-related change in VBM and change in route vs. scrambled identification accuracy (r(24)=-.308, p=.125).

There was no correlation between training-related change in VBM and change in specific route identification accuracy (r(24)=-.261, p=.198).

There was no correlation between training-related change in VBM and change in echo vs. no echo identification accuracy (r(24)=-.192, p=.347).

There was no correlation between training-related change in VBM and age (r(24)=.083, p=.687).

## V1_R

There was a significant effect of group (F(1,23)=6.991, p=.015, η_p_^2^ =.233), with SPs having higher grey matter density (adjusted mean = .360) than BPs (adjusted mean = .317). There was no effect of training (F(1,23)=.758, p=.393) and no interaction (F(1,23)=1.172, p=.290).

There was no correlation between training-related change in VBM and change in route vs. scrambled identification accuracy (r(24)=-.071, p=.730).

There was no correlation between training-related change in VBM and change in specific route identification accuracy (r(24)=-.175, p=.394).

There was no correlation between training-related change in VBM and change in echo vs. no echo identification accuracy (r(24)=-.100, p=.626). There was no correlation between training-related change in VBM and age (r(24)=.037, p=.857).

## OPA_L

There was a significant effect of group (F(1,23)=7.683, p=.011, η_p_^2^ =.250), with SPs having higher grey matter density (adjusted mean = .453) than BPs (adjusted mean = .383). There was no effect of training (F(1,23)=.110, p=.743) and no interaction (F(1,23)=.034, p=.855).

There was a significant positive correlation between training-related change in VBM and change in route vs. scrambled identification accuracy (r(24)=.393, p=.047).

There was no correlation between training-related change in VBM and change in specific route identification accuracy (r(24)=.039, p=.850).

There was no correlation between training-related change in VBM and change in echo vs. no echo identification accuracy (r(24)=-.190, p=.353).

There was no correlation between training-related change in VBM and age (r(24)=-.143, p=.487).

## OPA_R

There was no significant effect of group (F(1,23)=2.725, p=.112), no effect of training (F(1,23)=3.602, p=.070), and no a significant interaction (F(1,23)=2.586, p=.121).

There was no correlation between training-related change in VBM and change in route vs. scrambled identification accuracy (r(24)=.017, p=.936).

There was no correlation between training-related change in VBM and change in specific route identification accuracy (r(24)=.017, p=.936).

There was no correlation between training-related change in VBM and change in echo vs. no echo identification accuracy (r(24)=-.090, p=.663).

There was no correlation between training-related change in VBM and age (r(24)=-.156, p=.445).

# Additional supplemental figures S6 – S11


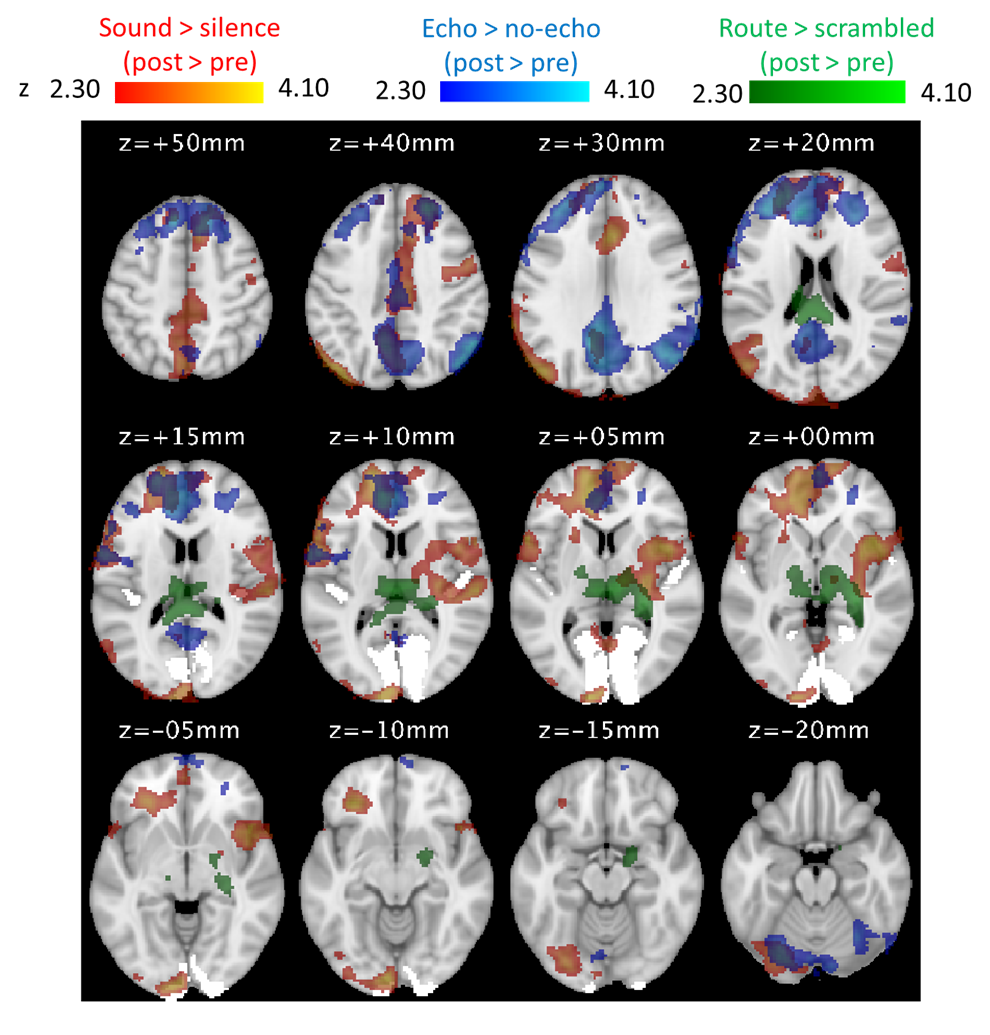


**Figure S6** Activation maps showing the effect of training (SPs only, with cluster thresholding) on each contrast displayed on the MNI152 standard-space template. The red maps show where activation was greater at post-training relative to pre-training for the sound vs silence contrast (z > 2.3). The blue maps show the same for the echo vs. no-echo contrast, and the green maps for the route vs. scrambled. Areas V1 and A1 are highlighted in white. Orientation of the images is in neurological convention (i.e. left is left).

**
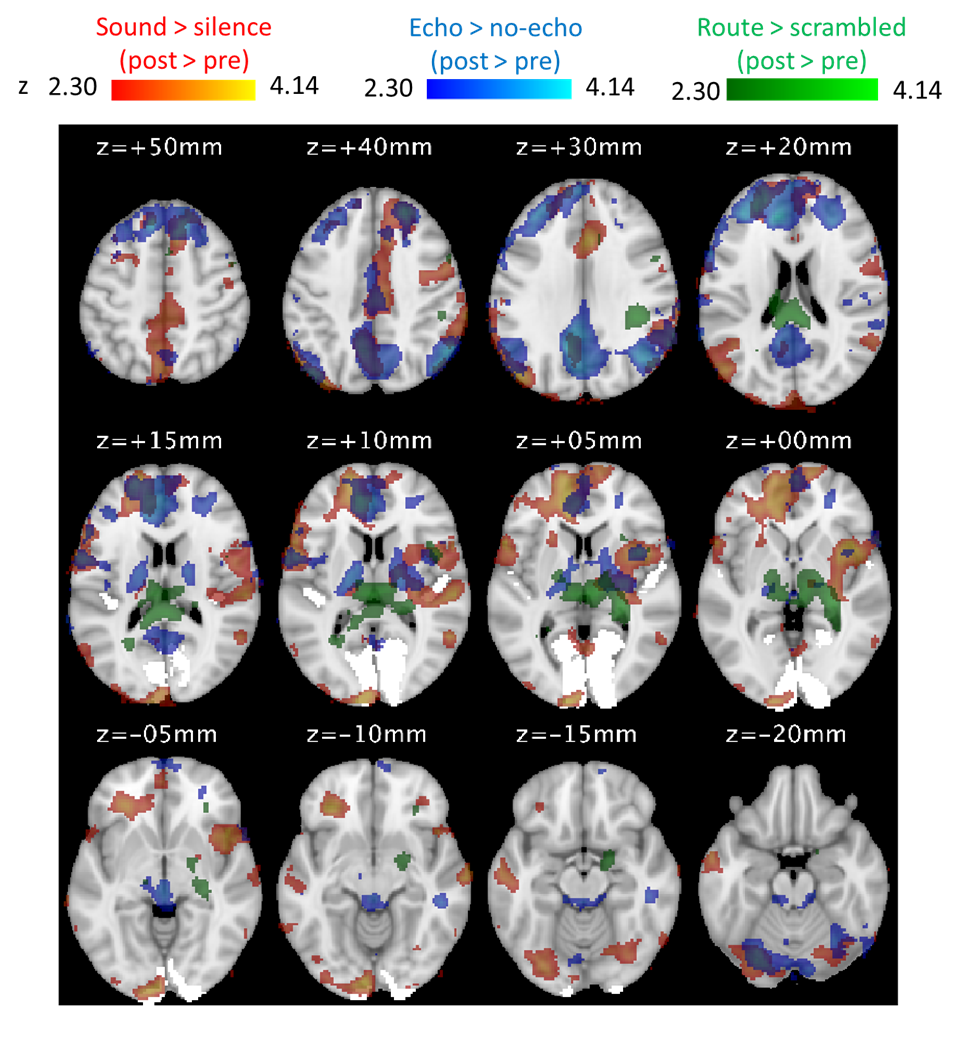
**

**Figure S7** Activation maps showing the effect of training (SPs only, without cluster thresholding) on each contrast displayed on the MNI152 standard-space template . The red maps show where activation was greater at post-training relative to pre-training for the sound vs silence contrast (z > 2.3). The blue maps show the same for the echo vs. no-echo contrast, and the green maps for the route vs. scrambled. Areas V1 and A1 are highlighted in white. Orientation of the images is in neurological convention (i.e. left is left).

**
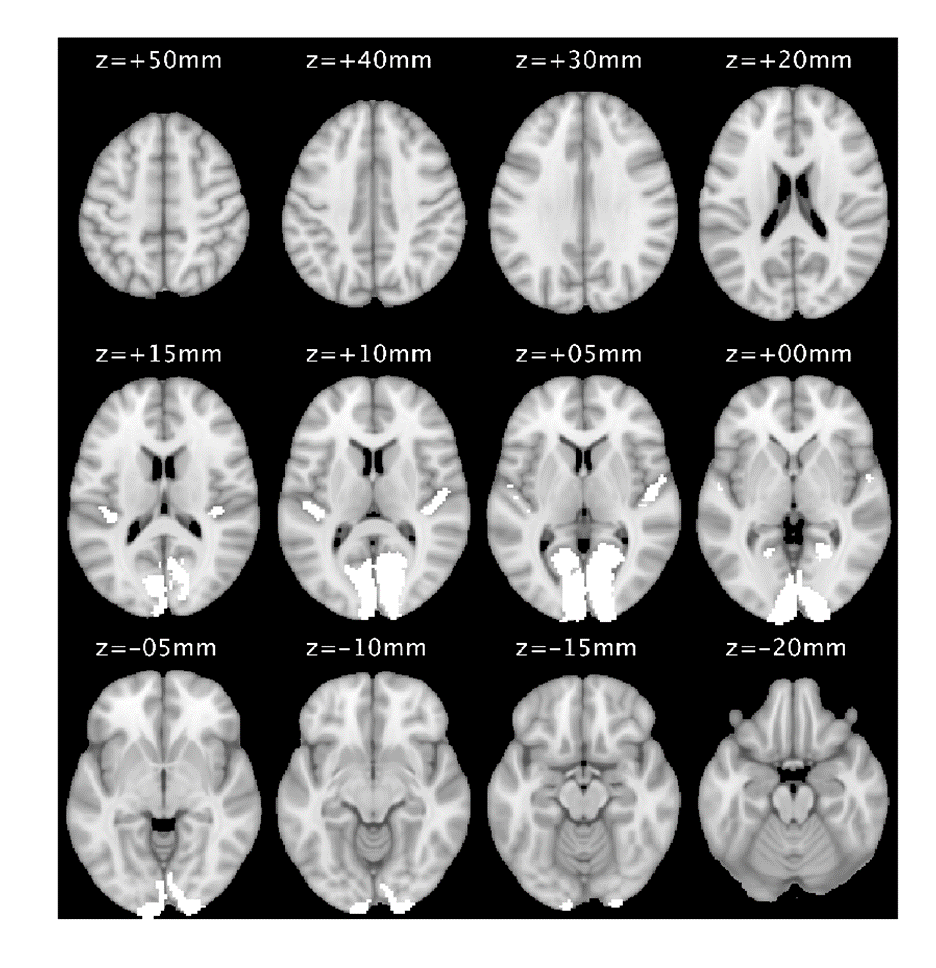
**

**Figure S8** Activation maps showing the effect of training (BPs only, with cluster thresholding) on each contrast displayed on the MNI152 standard-space template. There was no significant activation for any of the contrasts. Areas V1 and A1 are highlighted in white. Orientation of the images is in neurological convention (i.e. left is left).

**
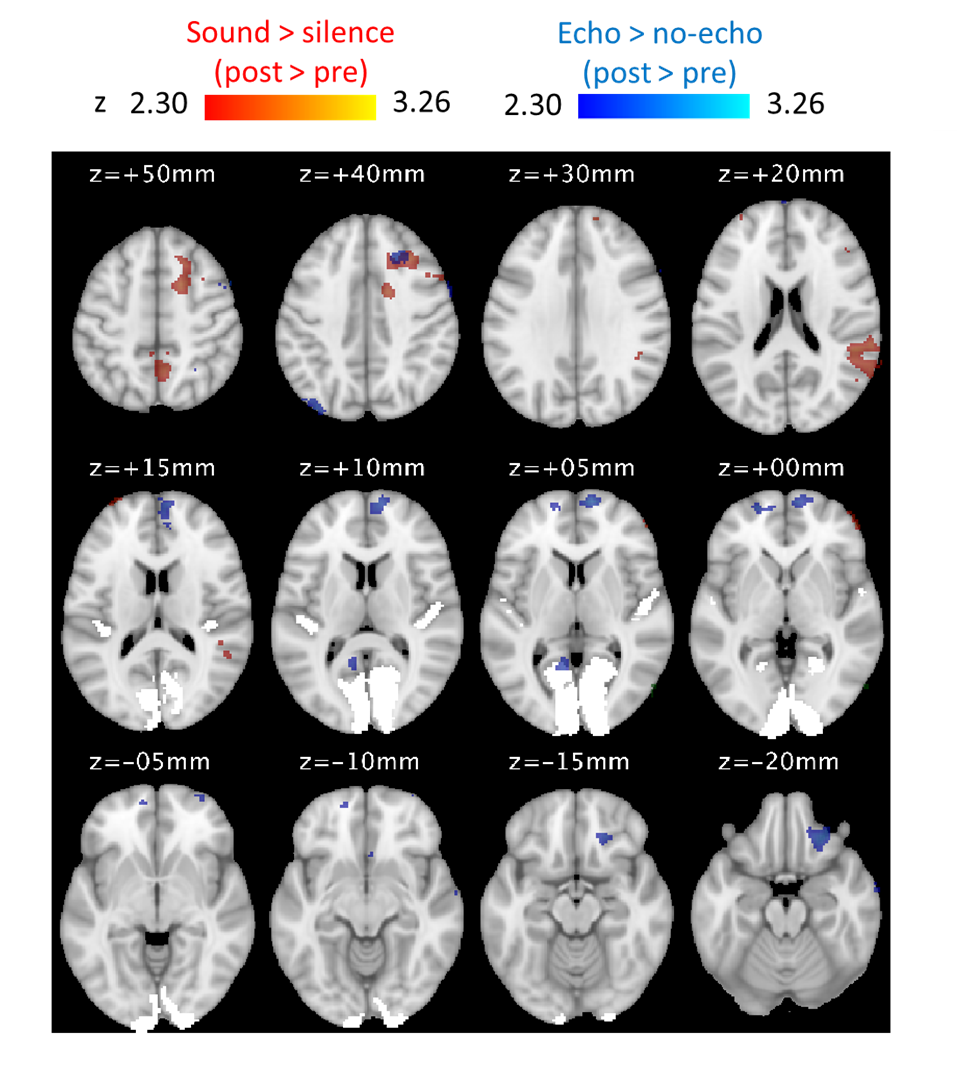
**

**Figure S9** Activation maps showing the effect of training (BPs only, without cluster thresholding) on each contrast displayed on the MNI152 standard-space template. The red maps show where activation was greater at post-training relative to pre-training for the sound vs silence contrast (z > 2.3). The blue maps show the same for the echo vs. no-echo contrast. There was no significant activation for the route vs. scrambled contrast. Areas V1 and A1 are highlighted in white. Orientation of the images is in neurological convention (i.e. left is left).


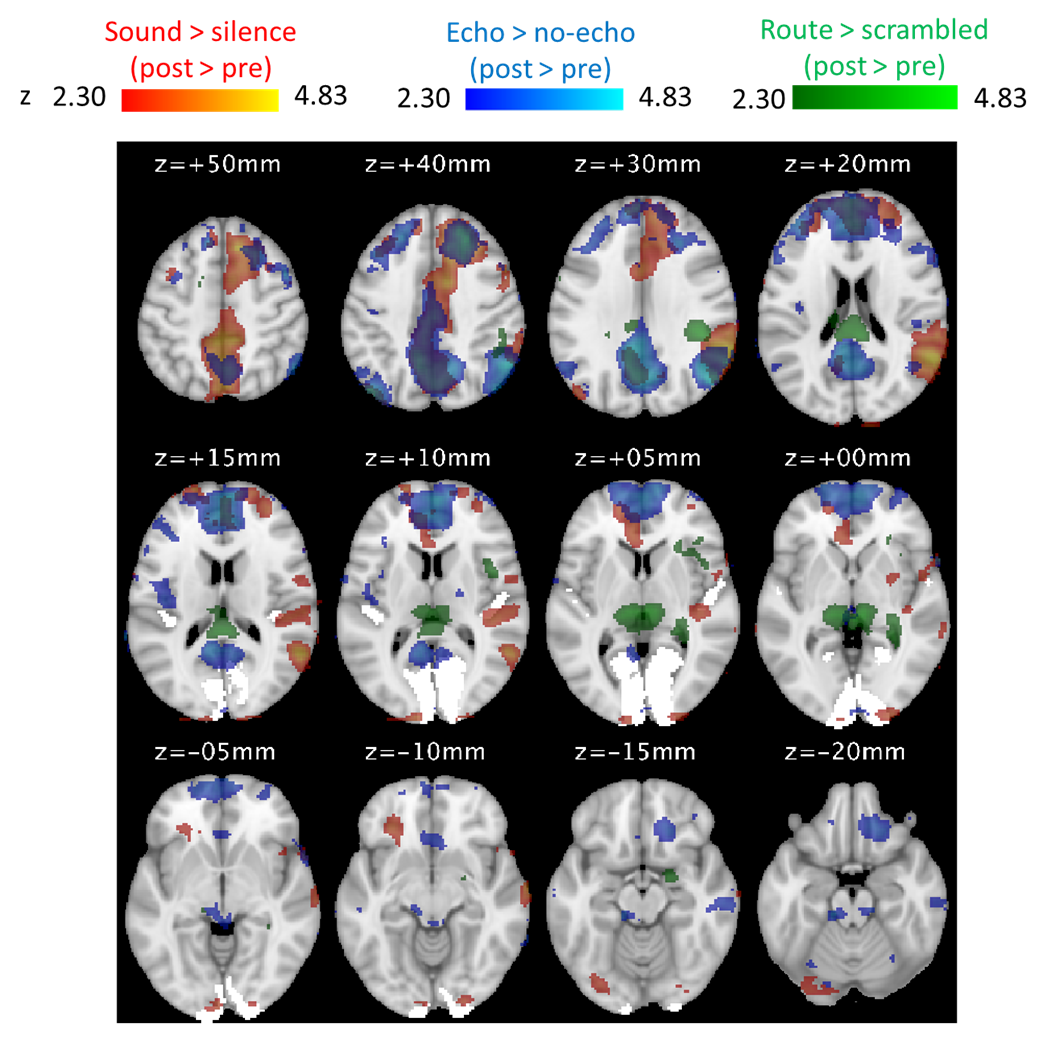


**Figure S10** Activation maps showing the effect of training (BPs and SPs as one group, without cluster thresholding) on each contrast displayed on the MNI152 standard-space template. The red maps show where activation was greater at post-training relative to pre-training for the sound vs silence contrast (z > 2.3). The blue maps show the same for the echo vs. no-echo contrast, and the green maps for the route vs. scrambled. BPs and SPs were entered as a single group in this analysis. Areas V1 and A1 are highlighted in white. Orientation of the images is in neurological convention (i.e. left is left).


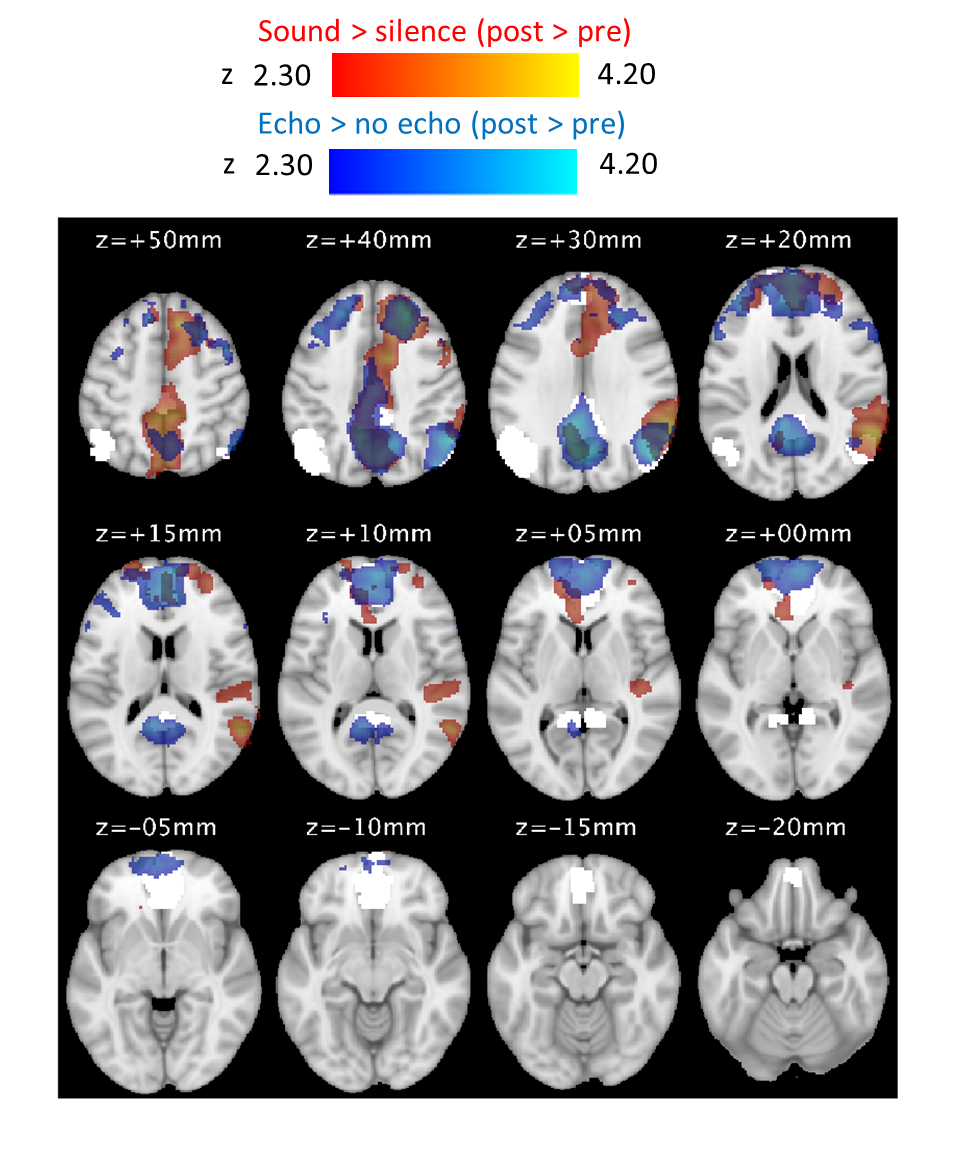


**Figure S11.** Same as Fig 7 in main text, but with the DMN shown with white mask overlay (after smoothing and binarization). DMN mask from Wang et al (2020). The mask was smoothed with a Gaussian kernel of .25 mm and then binarized.

# References

Norman, L. J., Dodsworth, C., Foresteire, D., & Thaler, L. (2021). Human click-based echolocation: Effects of blindness and age, and real-life implications in a 10-week training program. *PloS one*, *16*(6), e0252330. <https://doi.org/10.1371/journal.pone.0252330>

Wang, S., Tepfer, L.J., Taren, A.A. *et al.* (2020). Functional parcellation of the default mode network: a large-scale meta-analysis. *Sci Rep* **10**, 16096. <https://doi.org/10.1038/s41598-020-72317-8>
